# Effectiveness of the Components of a Digital Multiple Health Behavior Change Intervention Among Individuals Seeking Help Online (Coach): Factorial Randomized Trial

**DOI:** 10.2196/88881

**Published:** 2026-04-02

**Authors:** Joel Crawford, Katarina Åsberg, Jenny Blomqvist, Oskar Lundgren, Hanna Henriksson, Pontus Henriksson, Preben Bendtsen, Marie Löf, Marcus Bendtsen

**Affiliations:** 1 Department of Health, Medicine and Caring Sciences Linköping University Linköping, Östergötland Sweden; 2 Crown Princess Victoria Children’s Hospital Linköping University Hospital Linköping, Östergötland Sweden; 3 Unit for Strategic Healthcare Region Östergötland Linköping, Östergötland Sweden; 4 Motala Hospital Motala, Östergötland Sweden; 5 Department of Medicine Huddinge Karolinska Institutet Stockholm, Stockholm Sweden

**Keywords:** behavior change, digital interventions, multiple lifestyle, public health

## Abstract

**Background:**

Extant digital multiple health behavior change interventions have shown promise in various populations; however, evidence for a broader approach among the general population is lacking. Moreover, existing interventions often contain several components but are typically assessed as a whole, meaning it remains unclear to what extent individual components contribute to intervention effects and how they may interact to influence health outcomes.

**Objective:**

This study estimates the effects of 6 components of a digital health behavior change intervention on alcohol, diet, physical activity, and smoking outcomes among individuals searching for help online.

**Methods:**

A double-blind randomized factorial trial design with 6 two-level factors was used. Adults from the general public in Sweden who were seeking help to change their behaviors were recruited through web searches and social media. Participants were eligible if they were 18 years or older and had at least one health behavior classified as unhealthy. Effects of 6 components were estimated: screening/feedback, goal-setting/planning, motivation, skills/know-how, mindfulness, and self-authored SMS text messages. Primary outcomes were weekly alcohol consumption and frequency of heavy episodic drinking, average daily fruit and vegetable consumption, weekly moderate-to-vigorous physical activity, and 4-week point-prevalence smoking.

**Results:**

A total of 5419 individuals were randomized. Overall, the screening/feedback component was the most effective for changing health behaviors, along with goal-setting/planning and motivation to change. In particular, there was evidence that screening/feedback increased average daily portions of fruit and vegetables at 2 months (mean difference 0.17, compatibility interval [CoI] 0.09-0.25, probability of effect [POE] >99.9%) and at 4 months (mean difference 0.13, CoI 0.04-0.21, POE 99.9%) and reduced the frequency of heavy episodic drinking at 4 months (incidence rate ratio 0.91, CoI 0.81-1.03, POE 94.2%). Components also interacted to further improve health outcomes, most notably the combination of screening/feedback with motivation to change, which further increased fruit and vegetable consumption (2 months: mean difference 0.20, CoI 0.09-0.30, POE >99.9%; 4 months: mean difference 0.17, CoI 0.05-0.29, POE 99.8%).

**Conclusions:**

The results from this study contribute to the development of more effective interventions by providing novel insights into the effects of individual and pairwise components of complex digital health behavior change interventions.

**Trial Registration:**

ISRCTN Registry ISRCTN16420548; http://www.isrctn.com/ISRCTN16420548

**International Registered Report Identifier (IRRID):**

RR2-10.1136/bmjopen-2022-061024

## Introduction

Alcohol consumption, diet, physical activity, and smoking are leading factors determining general health and susceptibility to disease [1] and are prominent causes of noncommunicable diseases (eg, cardiovascular disease, respiratory disease, cancer, and diabetes) [1]. The increasing prevalence of chronic disease is one of the major challenges to global health, and unlike intrinsic etiologies of noncommunicable diseases, health behaviors are modifiable [2]. In light of this, the World Health Organization has set a charter for member states to lower the incidence of noncommunicable diseases by implementing measures to reduce engagement in health risk behaviors while increasing engagement in health promotion behaviors [2]. To achieve this, there is a need to develop and implement effective measures to help individuals improve their health behaviors.

In Sweden, individuals regularly engage in unhealthy behaviors; national surveys have shown that 40% of Swedish individuals drink at levels that indicate risky alcohol use, 66% fail to eat an adequate amount of vegetables, 32% report insufficient physical activity, 5.8% smoke daily, and 51% have obesity or are overweight [3,4]. Despite macro-level interventions aimed at curbing the incidence of unhealthy behaviors [5-7], the prevalence of these behaviors has remained consistent over the last 10 years, with the exception of tobacco smoking [4]. Overall, unhealthy lifestyle behaviors present a significant health burden for premature mortality and morbidity in Sweden, with 15% of all deaths and 2974 disability-adjusted life years (DALYs) attributed to tobacco use, 15% of all deaths and 2204 DALYs attributed to poor dietary behaviors, 5% of all deaths and 1292 DALYs attributed to alcohol use, and 2% of all deaths and 266 DALYs attributed to insufficient physical activity in 2019 (DALYs provided per 100,000 population) [8,9]. Thus, new means of intervening in the general public are warranted to avoid another decade of stagnating or increasing prevalence of unhealthy behaviors and subsequent maladies.

Current intervention efforts have relied on public health recommendations, national ad campaigns, and policy measures such as restrictions on availability and advertising [5-7]. While macro-level approaches do have some merit (ie, the ability to reach a significant portion of the populace), they are unable to address the barriers and intricacies of health behavior change; for example, they provide little support on how to change and do not consider that health behaviors often cluster and interact [10,11]. Evidence indicates that health behaviors consistently co-occur: for example, smoking and alcohol use, smoking and unhealthy diet, and physical inactivity and unhealthy diet [12]. A recent study conducted in Sweden found that 36% of adults had a co-occurrence of at least 2 unhealthy behaviors, with 10% having a co-occurrence of 3 behaviors and 2% having a co-occurrence of 4 behaviors [13]. The clustering of unhealthy behaviors significantly increases the risk of all-cause mortality and morbidity compared with a single behavior alone [14-16]. Current intervention efforts typically focus on a single behavior, meaning they are unable to target underlying clusters that considerably increase health risk [12]; hence, there is a need for interventions that can address the intricacies and barriers this presents to health behavior change.

One method to overcome issues with current prevention efforts is to offer digital support tools for those seeking help online. This approach has the potential to be widely disseminated while also addressing the intricacies of health behavior change. For instance, content can be tailored to personal dimensions of behavior and can also address barriers to health (eg, reducing stigma associated with seeking help) [17]. In addition, a digital approach may be particularly apt for Sweden due to the near-ubiquitous ownership of smartphones and the high prevalence of seeking help online—66% of individuals aged 16-84 years sought health advice online in 2023 [18]. While effectiveness trials for digital behavior change interventions targeting the Swedish general population have shown effectiveness for smoking cessation [19] and reducing alcohol consumption [20], there is currently a lack of digital interventions targeting multiple health behaviors [21]. Existing multiple health behavior change interventions have shown promise for various subpopulations, including university students, patients with a specific malady, and different professions [22-25]; however, evidence for a broader approach for the general population is lacking.

In addition, many current interventions use a sociocognitive framework to target modifiable determinants of health, such as intentions, attitudes, beliefs, self-efficacy, risk perceptions, motivation, planning, and expectancies [26]. Sociocognitive models of health and behavior change (eg, Theory of Planned Behavior, Health Action Process Approach, Capability-Opportunity-Motivation-Behavior [COM-B] model [27-29]) highlight that health behavior is seldom influenced by a single factor, but rather by a configuration of constructs that interact to influence health outcomes; they also indicate that targeting one factor without addressing deficits in others may not result in meaningful change [27,28]. Current interventions that target improvements in these determinants often use various components to instill change; however, they typically assess the effectiveness of the components as a whole [26]. This means that we are unsure to what degree separate components contribute to the effects of the interventions, how they interact to influence health outcomes, or whether certain components are effective only in conjunction with other commensurate components; for example, action planning may only be effective when self-efficacy or motivation is high [27,28]. Hence, gaining knowledge regarding the separate and interactive effects of intervention components would enable a more nuanced understanding of the effects of health behavior change interventions.

We conducted this study in response to the sparsity of studies investigating multiple health behavior change interventions for the general public and the need to better understand the effects of individual components of digital behavior change interventions. We used a factorial trial design to estimate the effects of 6 separate intervention components of a novel digital intervention targeting multiple health behaviors. The study was carried out among individuals seeking help online. Specifically, the study aimed to achieve the following:

- Estimate the effects of 6 individual intervention components on reducing alcohol use, improving dietary behaviors, increasing physical activity, and increasing smoking cessation.
- Estimate the pairwise interaction effects of the 6 intervention components on reducing alcohol use, improving dietary behaviors, increasing physical activity, and increasing smoking cessation.

## Methods

### Study Design

We conducted a factorial randomized trial (6 factors representing 6 components, C1-C6, each with 2 levels representing the absence or presence of the component) [30] to estimate the effects of a digital multiple health behavior change intervention among individuals seeking help online. The trial was an extension of the MoBILE (Mobile Health Multiple Lifestyle Behavior Interventions Across the Lifespan) research program [31] and was prospectively registered (ISRCTN16420548). A full protocol and analysis plan were published prospectively [32]. This report follows CONSORT (Consolidated Standards of Reporting Trials; Multimedia Appendix 1) guidelines [33], including extensions for factorial trials [34].

### Study Setting, Recruitment, and Participants

The target population was Swedish adults seeking help online to change their health behaviors. Participants were recruited through advertisements on Google (Alphabet Inc), Bing (Microsoft Corporation), Facebook (Meta Platforms, Inc), and websites related to lifestyle and behavior change. Individuals interested in participating sent an SMS text message to a dedicated phone number and received a reply with a link to a page containing informed consent materials (see Multimedia Appendix 2). All individuals who provided consent were then asked to complete a baseline survey, which was also used to assess eligibility (see Multimedia Appendix 3). To be included, individuals had to be 18 years of age or older and fulfill at least one of the conditions presented in Textbox 1. Note that, while there is no safe limit for alcohol consumption [35], we used Swedish national guidelines for alcohol to identify individuals who could benefit greatly from reducing their consumption.

As the study was conducted via participants’ smartphones, individuals without access to one were unable to take part. This also applied to those who did not have sufficient understanding of Swedish to comprehend the study materials.

**Criteria for inclusion.**

- **Weekly alcohol consumption:** Consumed 10 or more (for women) or 15 or more (for men) standard drinks of alcohol in the past week. In Sweden, a standard drink is defined as 12 g of pure alcohol.
- **Heavy episodic drinking:** Consumed 4 or more (for women) or 5 or more (for men) standard drinks of alcohol on a single occasion at least once in the past month.
- **Fruit and vegetables:** Consumed less than 500 g of fruit and vegetables on average per day in the past week.
- **Moderate-to-vigorous physical activity:** Spent less than 150 minutes on moderate-to-vigorous physical activity in the past week.
- **Smoking:** Smoked at least one cigarette in the past week.

### Interventions

The Coach intervention studied in this trial was a smartphone-based intervention comprising 6 components: (C1) screening/feedback, (C2) goal-setting/planning, (C3) motivation to change, (C4) skills/know-how, (C5) mindfulness, and (C6) self-authored SMS text messages. The components were informed by sociocognitive models of health [36] and have been included in interventions that have proven successful in facilitating behavior change [19,20]. While built on what is already known about digital health behavior change interventions, the novelty of the Coach intervention lies in its approach of including multiple behaviors within a single intervention. The intervention was designed so that each component could be presented to participants in a menu list, making it possible to add or remove components based on factorial conditions.

A core feature of the intervention was an SMS text message sent to participants each Sunday afternoon, containing a link and reminder to access the intervention materials. The intervention components, depending on factorial allocation, and weekly reminders were available to participants over 4 months. Table 1 provides an overview of each component, along with its relevant behavior change techniques [37]. Please see Multimedia Appendix 4 for full details of the intervention. As Coach was designed to be a multiple health behavior change intervention, all participants, regardless of their baseline screening, received intervention materials for alcohol, diet, physical activity, and smoking. There was no fixed pathway through the materials; rather, they were presented as a toolbox. Therefore, participants could engage with materials concerning different behaviors according to their own preferences and were not required to engage with materials for behaviors they did not find relevant at the time.

**Table 1:** Description of intervention components, conceptual framework, practical application, and factorial conditions.

Component	Conceptual framework	Practical application	Factorial conditions
Component 1: screening and feedback	*Self-monitoring* has been shown to be a potentially effective strategy for reducing excessive alcohol [37-40] consumption and promoting healthy eating and physical activity [41,42]. These include BCT^a^ 1.6 (Discrepancy between current behavior and goals), BCT 2.2 (Feedback on behavior), BCT 2.3 (Self-monitoring of behavior), and BCT 6.2 (Social comparison).	Every Sunday afternoon, participants received an SMS text message with a hyperlink to a questionnaire regarding their current health behaviors. Once the questionnaire was completed, participants received feedback on their current behaviors in comparison to national guidelines. Thereafter, users were given access to the rest of the components (depending on allocation).	When this component was absent, participants were not asked to respond to the screening questionnaire but were instead shown national guidelines without any personal feedback.
Component 2: goal-setting and planning	*Self-regulatory* skills and capacity via goal-setting and planning. Planning-related activities such as goal-setting, action planning, and practicing behavior have been shown to be effective in lifestyle behavior interventions [41,43-48]. These include BCT 1.1 (Goal-setting), BCT 1.2 (Problem solving), BCT 1.4 (Action planning), BCT 7.1 (Prompts/cues), and BCT 8.1 (Behavior practice/rehearsal).	This component allowed participants to set 1 or more goals for their future behavior. It included action planning for how they were to progress toward their goals, preparation for motivational struggles (coping planning), and strategies for rewarding themselves upon success. Participants could also create their own or accept ready-made challenges for the coming week, such as walking for 15 minutes each day or not drinking any alcohol the coming week. Reminders were sent via SMS text messages to participants about their goals and challenges throughout the week (up to 4 messages).	When absent, this component was not visible, and goal-setting reminders were not available.
Component 3: motivation	Digital health behavior change interventions have been shown to enhance s*elf-efficacy*; however, there is a lack of consensus across reviews as to which content works best to facilitate an increase in self-efficacy [49]. These include BCT 5.1 (Information about health consequences), BCT 9.1 (Credible source), BCT 9.2 (Pros and cons), and BCT 9.3 (Comparative imagining of future outcomes).	This component contained information and tools to increase participants’ awareness of their own motivation, encourage commitment, and boost self-efficacy. This included information on negative health consequences, costs induced by certain behaviors, and reflective tasks via texts, videos, and exercises. If participants chose, they could also activate motivational SMS text messages (derived from previously developed and evaluated interventions [50-56]) sent to them throughout the week (a maximum of 8-10 messages per week).	When absent, this component was not visible, and SMS text messages were not available.
Component 4: skills and know-how	Intervention content based on shaping knowledge, aimed at increasing an individual’s understanding and awareness to facilitate behavior change, has been shown to be effective [44,45,57,58]. These include BCT 4.1 (Instructions on how to perform a behavior), BCT 8.2 (Behavior substitution), BCT 8.3 (Habit formation), and BCT 8.7 (Graded tasks).	This component aimed to increase participants’ skills and know-how by providing concrete tips on how to initiate and maintain lasting changes in everyday life by repetition and substitution. Participants were given strategies (eg, how to say no to alcoholic beverages or how to introduce vegetables into their meals). If participants chose to, they could also activate SMS text messages with tips and know-how, which were sent to them throughout the week (a maximum of 8-10 messages per week).	When absent, this component was not visible, and SMS text messages were not available.
Component 5: mindfulness	The mindfulness exercises were based on previous research and considered to be evidence-based methods to improve the mental well-being of clinical populations, while effects on behavior change in nonclinical settings are less well-studied [59-63].	This component aimed to increase users’ awareness of their own lived experience and strengthen their capacity for a nonreactive, compassionate, and less stressful way of being in the world. Mindfulness exercises were offered to participants, including guided meditations.	When absent, this component was not visible, and guided meditations were not available.
Component 6: self-authored SMS text messages	SMS text message self-authorship is generally understudied in the literature but was included in an effective digital alcohol intervention [64].	This component allowed participants to self-author up to 3 SMS text messages and schedule them to be sent to themselves throughout the week at times of their choosing. For example, a participant could write an SMS text message reminding themselves to eat 2 fruits each day, avoid drinking on Wednesdays, or go for a walk with a friend.	When absent, this component was not visible, and the ability to self-author SMS text messages was not available.

^a^BCT: behavior change technique.

### Outcomes and Measures

Outcomes are listed in Textbox 2 and subsequently explained. All questionnaires used in the trial can be found in Multimedia Appendix 3.

Weekly alcohol consumption was assessed using a short-term recall method that asked participants to record the number of standard drinks consumed in the past week [65]. The frequency of heavy episodic drinking was assessed by asking participants to report how many times they consumed 4 or more (for women) or 5 or more (for men) standard drinks per session over the past month. These 2 outcomes are both part of the core outcome set for brief alcohol interventions [66,67].

Diet and physical activity were assessed using items based on a questionnaire from the Swedish National Board of Health and Welfare [21]. The items were adapted to include portion sizes (100 g of fruit or vegetables, respectively) and asked how many portions were consumed per day in the preceding week. Sugary drink consumption was assessed by asking participants to report the number of units (33 cl) of sugary drinks consumed in the past week. Moderate-to-vigorous physical activity (MVPA) was assessed with 2 items asking for the number of minutes spent in both moderate and vigorous activity in the past week; responses were summed to provide a total MVPA value. BMI was measured by asking participants to report their height (cm) and weight (kg).

Four-week point-prevalence smoking abstinence (no cigarettes in the past 4 weeks) was assessed using a binary item. Those reporting nonabstinence were asked to record the number of cigarettes smoked in the past week. These items were based on suggestions from the Society for Research on Nicotine [68].

Perceived stress was assessed using the short-form Perceived Stress Scale [69], and quality of life (QoL) was measured using Patient-Reported Outcomes Measurement Information System (PROMIS) Global 10 [70].

**Primary and secondary outcomes.**

**Primary outcomes**

- **Alcohol:** Weekly alcohol consumption; monthly frequency of heavy episodic drinking
- **Diet:** Average daily consumption of fruit and vegetables
- **Physical activity:** Weekly moderate-to-vigorous physical activity
- **Smoking:** Four-week point prevalence of smoking abstinence

**Secondary outcomes**

- Weekly consumption of sweets and snacks
- Weekly consumption of sugary drinks
- BMI
- Weekly number of cigarettes smoked
- Perceived stress
- Quality of life

### Follow-Up

Primary and secondary outcomes were assessed at 2 and 4 months after randomization. The 2-month follow-up facilitated the assessment of the immediate effects of the components, while the 4-month follow-up facilitated the estimation of effects after prolonged access. Additional longer follow-up intervals were not included to reduce participant burden, and because attrition was expected to be much higher the longer the time from baseline. An additional follow-up was conducted at 1 month after randomization to assess the proposed mediators of the intervention. Mediator analyses will be reported separately. Follow-ups were initiated by SMS text messages containing hyperlinks to questionnaires (see Multimedia Appendix 3). A total of 2 reminder SMS text messages were sent, 2 days apart, to nonresponders. If no response was received, participants were contacted by phone and asked to respond to the primary outcome measures only. A total of 5 call attempts were made.

### Randomization and Blinding

We used block randomization with random block sizes of 64 and 128 to allocate participants equally among factors. The process was automated, with the sequence computer-generated and the backend server allocating eligible participants after completion of baseline assessments. Neither research personnel nor participants were able to influence allocation. Research personnel were blinded to allocation throughout the trial. Participants were also blinded, as they had access to different components of the trial but were unaware of the other available conditions. All measurements, including baseline and follow-up, were conducted through digital questionnaires completed by participants independently on their smartphones, thereby preserving blinding during measurement. Attempts to collect data from nonresponders via smartphones introduced a risk of disclosure of allocation for some participants; however, research personnel were experienced and instructed to ask only about the outcome measures. In addition, given that there were 64 conditions in the trial, it would have been difficult for participants to inadvertently reveal their allocation.

### Statistical Analysis

#### Intention-to-Treat Analysis and Bayesian Multilevel Modeling

We conducted analyses keeping participants in the conditions to which they were randomly allocated (intention to treat). We estimated models using both available data and data with missing values imputed (multiple imputation with chained equations [71], with 100 datasets and 30 iterations using predictive mean matching). All statistical analyses were prespecified in the study protocol [32].

We contrasted the presence versus absence of components using multilevel regression models with covariates for time-by-component interactions and participant-level adaptive intercepts. We also estimated models with pairwise interactions between components. All models were adjusted for the presence or absence of all components, as well as baseline measures of age, sex, importance, confidence, and know-how. Primary outcomes, perceived stress, and weekly number of cigarettes smoked were additionally adjusted for their respective baseline measures. BMI, sugary drink consumption, and sweets and snacks were adjusted for baseline MVPA minutes per week and average daily intake of fruit and vegetables. QoL was adjusted for perceived stress at baseline.

Bayesian inference was used to estimate the parameters of the models [72]. We used standard normal priors for all covariates and random intercepts to reflect a conservative view of effect magnitudes; thus, the priors induced shrinkage to protect against spurious findings. For each coefficient of interest, we report the marginal posterior probability of effect (POE), using the median as a point estimate of the magnitude of the effect. We also report 95% compatibility intervals (CoIs), defined by the 2.5% and 97.5% percentiles of the posterior distributions.

#### Primary and Secondary Outcomes

We analyzed primary outcomes among participants who fulfilled each respective inclusion criterion; for example, the MVPA outcome was analyzed among those with less than 150 minutes of MVPA in the past week at baseline. Secondary outcomes were analyzed among all participants, except for the number of cigarettes smoked in the past week, which was analyzed among baseline smokers who continued to smoke.

All count variables, including total weekly alcohol consumption, frequency of heavy episodic drinking, consumption of sugary drinks, sweets and snacks, and cigarettes smoked per week, were analyzed using negative binomial regression models. Average daily intake of fruit and vegetables, minutes of MVPA per week, BMI, QoL, and perceived stress were analyzed using linear regression. Point-prevalence smoking abstinence was analyzed using logistic regression.

#### Ancillary Analyses

We studied attrition by estimating the odds of responding to follow-up, conditional on baseline characteristics and the presence or absence of the 6 components (factors), using logistic regression. We estimated 1 model without interaction terms and a second model including interaction terms between factors and baseline characteristics. We used Cauchy priors to promote a parsimonious model (centered at 0 with a standard normal hyperprior for scale). We also modeled primary outcome data conditional on the number of attempts to collect follow-up. Under the assumption that late responders are more similar to nonresponders than early responders are, any association between the number of attempts and outcomes may indicate systematic attrition.

#### Post Hoc Analyses

To investigate whether the number of components to which participants had access affected outcomes, we post hoc modeled outcomes conditional on the number of components. The same model specifications were used as in the primary analyses, with dummy variables for components replaced by a variable for component count.

### Sample Size

We used a Bayesian sequential design to monitor when to stop recruitment [73]. As 4-month follow-up data became available, we modeled each of the primary outcomes according to the analysis plan, and effect estimates for each component were assessed against targets for effect, harm, and futility (see protocol for details of the criteria [32]). We anticipated that recruitment would not last more than 12 months; however, we allowed recruitment to continue for a total of 24 months and stopped recruitment at that point, despite targets not being met for all outcomes.

### Ethical Considerations

Ethical approval was received on August 11, 2021, from the Swedish Ethical Review Authority (approval Dnr 2021-02855). All participants were provided with study information and gave informed consent before responding to the baseline questionnaire and subsequently being randomized. Participants received no compensation for participating in the trial. Participants’ phone numbers were encrypted and stored in the study database to link baseline and follow-up data. At project completion, the encrypted phone numbers will be deleted and the data will be anonymized.

## Results

### Overview

**Figure 1:**
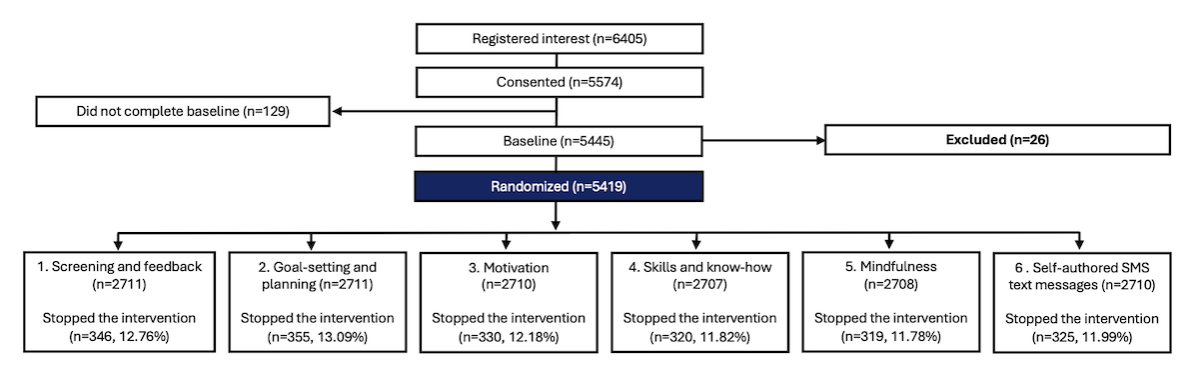
CONSORT (Consolidated Standards of Reporting Trials) participant flow diagram—sign-up and randomization.

**Figure 2:**
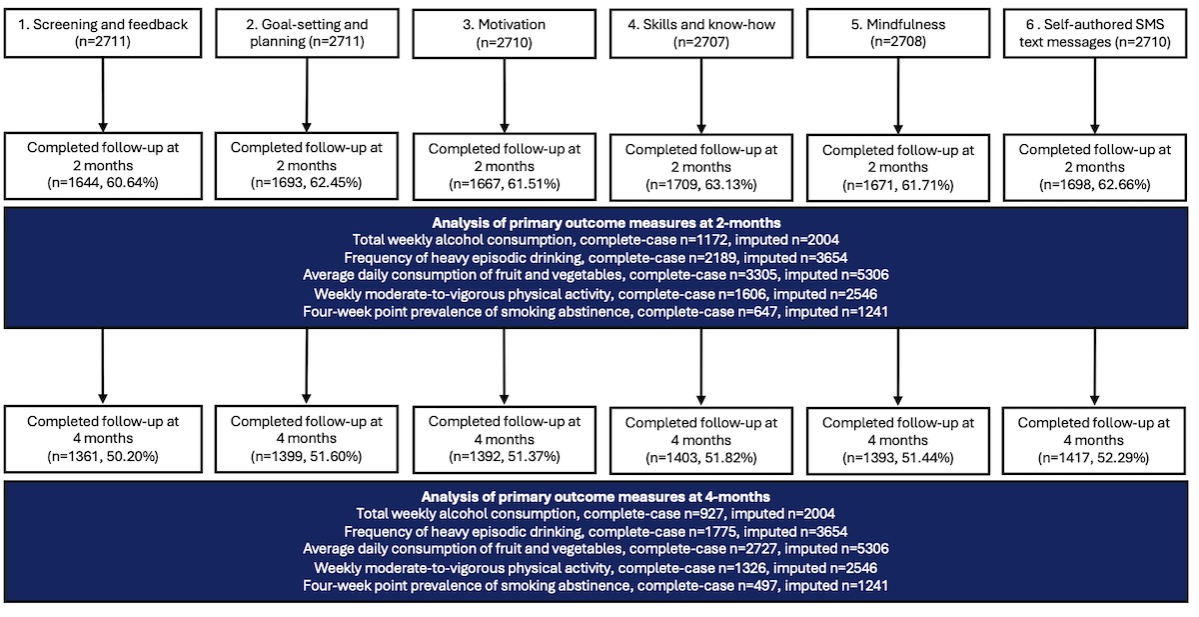
CONSORT (Consolidated Standards of Reporting Trials) participant flow diagram—follow-up.

A CONSORT participant flow diagram is presented in Figure 1 (sign-up and randomization) and Figure 2 (follow-up). Table 2 provides an overview of participants’ baseline characteristics. Between October 19, 2021, and October 19, 2023, a total of 6405 individuals showed interest in the study, of whom 5574 consented. A total of 5445 completed the baseline questionnaire, revealing that 26 participants were not eligible for inclusion. The remaining 5419 individuals were randomized. Among the 5419 participants, 650 (11.99%) decided to stop the reminder SMS text messages before the 16-week program had ended. We did not investigate the reasons why individuals chose to stop the reminders; however, all randomly allocated participants were sent follow-up questionnaires. Primary outcome data were available for 3371 (62.21%) participants at the 2-month follow-up interval and for 2786 (51.41%) at the 4-month interval.

**Table 2:** Baseline characteristics of randomly allocated participants.

Characteristics		Total (N=5419)	Screening/feedback (n=2711)	Goal-setting/ planning (n=2711)	Motivation (n=2710)	Skills/know-how (n=2707)	Mindfulness (n=2708)	Self-authored SMS text message (n=2710)
**Sex, n (%)**								
	Women	4301 (79.37)	2140 (78.94)	2141 (78.97)	2151 (79.37)	2172 (80.24)	2164 (79.91)	2136 (78.82)
	Men	1118 (20.63)	571 (21.06)	570 (21.03)	559 (20.63)	535 (19.76)	544 (20.09)	574 (21.18)
Age (years), mean (SD)		50 (11.7)	49.8 (11.8)	50 (11.7)	50.6 (11.6)	50 (11.4)	50.4 (11.6)	51 (11.5)
**Alcohol, mean (SD)**								
	Total weekly alcohol consumption (standard drinks)	8.89 (8.94)	8.88 (8.99)	8.91 (9.00)	8.54 (8.58)	9.04 (8.67)	8.77 (8.97)	9.48 (8.87)
	Frequency of heavy episodic drinking	4.80 (7.10)	4.77 (7.07)	4.91 (7.27)	4.54 (6.57)	4.76 (6.57)	4.46 (6.19)	4.22 (5.54)
**Smoking**								
	Number of smokers, n (%)	1240 (22.88)	636 (23.46)	626 (23.09)	632 (23.32)	633 (23.38)	615 (22.71)	636 (23.47)
	Number of cigarettes last week, mean (SD)	15.27 (38.0)	15.28 (37.5)	15.04 (38.2)	16.01 (39.9)	15.26 (38.2)	11.89 (34.2)	11.72 (32.3)
**Physical activity**								
	Moderate-to-vigorous physical activity (minutes), mean (SD)	220.4 (226.9)	226.5 (231.4)	208.7 (221.5)	222.0 (232.1)	225.3 (220.3)	210.2 (201.2)	215.3 (234.2)
**Dietary behavior**								
	Average daily fruit and vegetable consumption (portions), mean (SD)	1.49 (1.17)	1.53 (1.18)	1.39 (1.12)	1.47 (1.18)	1.47 (1.17)	1.61 (1.17)	1.34 (1.04)
**Stress**								
	Self-perceived stressª, mean (SD)	7.62 (2.80)	7.60 (2.77)	7.59 (2.89)	7.67 (2.92)	7.82 (2.69)	7.51 (2.65)	7.55 (2.44)
**Psychosocial measures, median (IQR)**								
	Importance^b^	10 (8-10)	10 (8-10)	10 (8-10)	10 (8-10)	10 (8-10)	10 (8-10)	10 (9-10)
	Confidence^b^	7 (5-8)	7 (5-8)	7 (5-8)	7 (5-8)	7 (5-8)	6 (5-8)	6 (5-8)
	Know-how^b^	6 (5-8)	6 (5-8)	6 (5-8)	6 (5-8)	6 (5-8)	6 (5-8)	6 (4-8)

^a^The 4-item Perceived Stress Scale with total scores ranging from 0 to 16.

^b^Single item with 1-10 response options.

### Attrition

Results from the attrition analyses can be found in Multimedia Appendices 5 and 6. In summary, we found evidence that older participants were more likely to respond to follow-up than younger participants at both follow-up intervals. In addition, those with more frequent episodes of heavy drinking and those who smoked more at baseline were less likely to respond to follow-up. Notably, these associations were not moderated by differential access to components.

We found evidence suggesting that late responders differed from early responders with respect to fruit and vegetable consumption, MVPA, and smoking. The evidence also suggested that these associations were moderated by differential access to components. Under the assumption that late responders are more similar to nonresponders than early responders are, this provides evidence of systematic attrition.

### Primary and Secondary Outcomes

#### Overview

Multimedia Appendix 7 contains tables reporting the main effects and 2-way interactions for the intervention components with available data, whereas Multimedia Appendix 8 contains the corresponding effect estimates with missing data imputed. Here, we highlight findings for which the evidence of effects was strongest, quoting estimates from analyses of available data.

#### Primary Outcomes

##### Total Weekly Alcohol Consumption

There was no consistent evidence of effects from any of the components on weekly alcohol consumption, either individually or in combination. The relatively strongest evidence was found for an effect of screening/feedback (C1) at 4 months (incidence rate ratio [IRR] 0.94, CoI 0.83-1.07, POE 82.4%). In addition, there was some evidence of an effect of goal-setting/planning (C2) at 2 months (IRR 0.95, CoI 0.85-1.06, POE 81.8%), but this effect was not observed at 4 months. The evidence suggested that combining screening/feedback (C1) with skills/know-how (C4) resulted in even lower alcohol consumption at 4 months (IRR 0.92, CoI 0.76-1.11, POE 81.4%). These findings persisted when imputing missing data.

##### Frequency of Heavy Episodic Drinking

Among individual components, the relatively strongest evidence for reduced frequency of heavy episodic drinking was found for screening/feedback (C1) at 4 months (IRR 0.91, CoI 0.81-1.03, POE 94.2%). However, there was considerable evidence of harm from multiple combinations of components at the 4-month follow-up interval. For instance, combining goal-setting/planning (C2) with motivation (C3) (IRR 1.14, CoI 0.97-1.31, POE 93.4%), mindfulness (C5) (IRR 1.15, CoI 0.97-1.37, POE 94.6%), or self-authored SMS text messages (C6) (IRR 1.17, CoI 0.98-1.38, POE 96.3%) was associated with increased frequency of heavy episodic drinking. These findings persisted in analyses with missing data imputed.

##### Daily Portions of Fruit and Vegetables

Individually, there was strong evidence that screening/feedback (C1) increased daily portions of fruit and vegetables at 2 months (mean difference 0.17, CoI 0.09-0.25, POE >99.9%) and at 4 months (mean difference 0.13, CoI 0.04-0.21, POE 99.9%). There was also evidence that combining screening/feedback (C1) with other components increased effects, most prominently when combined with motivation (C3) at 2 months (mean difference 0.20, CoI 0.09-0.30, POE >99.9%) and at 4 months (mean difference 0.17, CoI 0.05-0.29, POE 99.8%). There was evidence that goal-setting/planning (C2) decreased fruit and vegetable consumption at 4 months (mean difference −0.08, CoI −0.16 to 0.00, POE 97.1%), and even more so when combined with skills/know-how (C4) (mean difference −0.14, CoI −0.26 to −0.02, POE 99.1%). These findings persisted in the imputed data.

##### Weekly MVPA

There was no consistent evidence of effects from any of the components, nor from combinations of components, on MVPA. The relatively strongest evidence in the available-data analyses suggested that mindfulness increased minutes of MVPA at 4 months (mean difference 10.3, CoI −11.3 to 32.1, POE 82.3%), and even more so when combined with screening/feedback (C1), goal-setting/planning (C2), and motivation (C3). However, in the imputed analyses, there was no evidence of these effects.

##### Smoking

There were several individual components for which there was evidence of effects on the point prevalence of smoking cessation in the available-data analyses. Notably, motivation (C3) increased the odds of smoking cessation at 4 months (odds ratio [OR] 2.4, CoI 0.98-5.99, POE 97.2%), whereas mindfulness (C5) decreased the odds of smoking cessation at 2 months (OR 0.35, CoI 0.15-0.78, POE 99.5%). These findings persisted in the imputed data, whereas evidence for other individual component effects did not. Evidence for effects of combined components was most consistent across available-data and imputed analyses for the combination of motivation (C3) and self-authored SMS text messages (C6) at 4 months (OR 2.42, CoI 0.70-8.72, POE 91.9%), and the combination of mindfulness (C5) and self-authored SMS text messages (C6) at 2 months (OR 0.23, CoI 0.07-0.71, POE 99.4%). The harmful effects of mindfulness that were prominent at 2 months were less evident at 4 months. There was no consistent evidence suggesting that any of the components, individually or in combination, affected the number of cigarettes smoked per week among those who continued smoking.

#### Secondary Outcomes

##### Weekly Sugary Drink Consumption

There was evidence that screening/feedback (C1) initially reduced weekly sugary drink consumption both individually (IRR 0.87, CoI 0.72-1.10, POE 92.2%) and in combination with skills/know-how (C4) and mindfulness (C5). However, there was no strong evidence that these effects persisted at 4 months, and findings were attenuated in the imputed analyses. By contrast, evidence based on available-data and imputed analyses suggested that skills/know-how (C4) reduced sugary drink consumption at 4 months (IRR 0.85, CoI 0.70-1.05, POE 93.8%), and increasingly so when combined with goal-setting/planning (C2) and motivation (C3). There was also evidence that combining motivation (C3) and self-authored SMS text messages (C6) increased sugary drink consumption at 2 months (IRR 1.22, CoI 0.92-1.59, POE 91.6%); however, there was no evidence of this harmful effect at 4 months.

##### Weekly Portions of Candy and Snacks

There was evidence that screening/feedback (C1) reduced weekly portions of candy and snack consumption at 2 months (IRR 0.93, CoI 0.85-1.02, POE 95.1%) and 4 months (IRR 0.92, CoI 0.83-1.01, POE 96.0%). In addition, there was evidence that motivation (C3) also reduced consumption at 4 months (IRR 0.85, CoI 0.77-0.94, POE 99.9%). Combining screening/feedback (C1) and motivation (C3) led to even greater reductions at 4 months (IRR 0.77, CoI 0.67-0.89, POE >99.9%). There was also evidence of increased effects when screening/feedback (C1) was combined with goal-setting/planning (C2), as well as with skills/know-how (C4). These findings persisted in the imputed-data analyses.

##### BMI

The relatively strongest evidence of effects on BMI was found for mindfulness (C5), which reduced BMI at both 2 months (mean difference −0.24, CoI −0.70 to 0.18, POE 86.4%) and 4 months (mean difference −0.23, CoI −0.69 to 0.20, POE 85.0%). There was also evidence that when mindfulness (C5) was combined with skills/know-how (C4), BMI was further reduced at both 2 months (mean difference −0.41, CoI −1.00 to 0.19, POE 90.7%) and 4 months (mean difference −0.47, CoI −1.07 to 0.14, POE 92.9%). These findings persisted in the imputed-data analyses.

##### Perceived Stress

The most consistent evidence of effects on perceived stress scores was found for the mindfulness (C5) component, both at 2 months (mean difference −0.17, CoI −0.37 to 0.03, POE 95.4%) and at 4 months (mean difference −0.22, CoI −0.44 to 0.0, POE 97.4%). Evidence of additional benefit at the 4-month follow-up interval was found when mindfulness was combined with screening/feedback (C1), as well as with motivation (C3). These findings persisted in the imputed analyses. There was also evidence that skills/know-how (C4) reduced perceived stress scores at 2 months (mean difference −0.25, CoI −0.45 to −0.05, POE 99.2%); however, the evidence suggested that the benefit did not persist at 4 months.

##### Quality of Life

There was no strong evidence of effects of the components, either individually or in combination, on QoL. Relatively, the strongest evidence was found for motivation (C3), which increased QoL scores both individually (mean difference 0.26, CoI −0.31 to 0.84, POE 81.7%) and in combination with skills/know-how (C4) (mean difference 0.54, CoI −0.28 to 1.35, POE 90.5%). These findings persisted but were attenuated in the imputed-data analyses.

#### Post Hoc Analyses

In Multimedia Appendix 9, we present estimates of the effects of the number of components to which participants had access on primary and secondary outcomes. The relatively strongest evidence for effects was found for increasing component count with regard to fruit and vegetable consumption and perceived stress at the 2-month follow-up. There was also evidence of reduced candy and snack consumption with increased component count at the 4-month follow-up interval.

## Discussion

### Primary Findings

This study used a factorial design to estimate the effectiveness of intervention components on multiple health behaviors, along with proxies for health and well-being. The results highlight novel findings regarding both beneficial and harmful effects of specific components, as well as how these components interact to affect health outcomes.

Screening/feedback (C1) was most consistent in reducing alcohol consumption, in terms of both weekly consumption and heavy episodic drinking. Further reductions in weekly consumption were observed when combined with skills/know-how (C4). These findings are consistent with extant digital alcohol interventions focused on screening and feedback, which have been shown to reduce alcohol consumption [20,39,74]. In addition, evidence from a study examining the mediated effects of an alcohol reduction app, “Drink Less,” found that tracking (ie, self-monitoring behavior) mediated the effects of the intervention [75]. However, in this study, notable evidence of harm, in terms of increased frequency of heavy episodic drinking, was found for combinations of components, particularly those including self-authored SMS text messages. We can only speculate why this was the case, but conflicting priorities in behavior change may have partially contributed to these harms. Notably, the intervention content was based on a digital alcohol intervention previously shown to be effective among people seeking help online [20]; however, when this content was combined with materials targeting other behaviors, it may not have been sufficiently tailored to individuals who typically engage in heavy episodic drinking. This highlights the need for caution when offering multiple behavior interventions to individuals whose primary focus should be on reducing heavy episodic drinking rather than improving overall lifestyle.

For smoking cessation, motivation (C3) was the most effective component, along with its interaction with other components, most notably with self-authored SMS text messages. Motivation reflects the importance of quitting smoking, suggesting that motivation to change is influenced by the perceived value of the outcome; the importance of the decision to quit is an integral part of cessation [76]. Evidence indicates that perceived benefits of cessation are associated with increased motivation to quit [77], and that positive abstinence outcome expectancies are associated with greater intentions to quit [78]. An analysis of individualized effects from a recent smoking cessation trial in Sweden showed that those who considered it highly important to quit reported better outcomes than those who considered it less important [79]. By contrast, we found that mindfulness (C5) was harmful in the short term, reducing the odds of smoking cessation at 2 months. Although speculative, many smokers use cigarettes as a stress reliever, and initiating a mindfulness program may initially heighten awareness of previously suppressed feelings, which can feel overwhelming. Previous research has shown that mindfulness interventions may be associated with increased stress during an initial phase before new coping strategies are established [80,81]. Furthermore, this study used self-directed practice, which lacks the emotional support of a group setting and guidance from an experienced teacher. Thus, individuals who smoked may have been more likely to continue smoking in the short term as a coping strategy. Notably, these harmful effects were not observed at the 4-month follow-up.

The screening/feedback component was effective in improving dietary outcomes, including increasing fruit and vegetable consumption, decreasing candy and snack consumption, and, to a lesser extent, reducing sugary drink consumption. This finding is consistent with a review by König et al [82], which reported that feedback and self-monitoring of dietary behavior may be key to intervention effectiveness. Nonetheless, the literature has yet to establish a dose-response relationship between feedback effects and engagement. Some studies suggest that daily tracking and feedback are effective [83,84], whereas others indicate that this may be too intrusive and therefore less effective [82]; by contrast, the current trial demonstrated that weekly screening and feedback can improve outcomes. Future research is needed to establish consensus on the appropriate level of screening and feedback, potentially by examining how self-perception interacts with external feedback to influence behavior [85]. Screening/feedback also interacted with various components to further improve dietary outcomes, most prominently with motivation (C3). Goal-setting/planning (C2) was also effective in improving candy and snacks consumption, supporting findings that demonstrate action planning and goal-setting as effective approaches [45,86,87]. The impact on dietary outcomes may be explained by how components interact to influence factors necessary for change, as outlined in various sociocognitive models of health [29,88,89]. For example, motivation may increase intentions, goal-setting and planning may help translate intentions into action, and feedback on performance may further enhance efficacy. This suggests that changing dietary behaviors may be best achieved through interventions that target motivation, facilitate the translation of intentions into action, and provide performance feedback. An extant intervention incorporating these components has been shown to improve fruit and vegetable consumption [90], while the current results suggest they may also help reduce consumption of calorie-dense foods and sugary drinks.

There was no consistent evidence that the intervention components, either individually or in pairwise combinations, affected physical activity. This is not consistent with existing literature suggesting that goal-setting and planning are effective intervention approaches for increasing engagement in physical activity [91,92]. There was some evidence indicating that mindfulness was effective in increasing physical activity, which aligns with prior evidence highlighting the effectiveness of this approach [93]. Nonetheless, in the imputed analyses, there was limited evidence of effects on physical activity. There was, however, relatively stronger evidence that mindfulness reduced BMI, supporting findings across trials that highlight the efficacy of mindfulness-based interventions for weight loss [94]. Evidence for skills/know-how (C4) also suggested effectiveness in reducing BMI, consistent with studies demonstrating the efficacy of skills training for weight loss [95,96]. Furthermore, an interaction between mindfulness and skills/know-how resulted in the largest reduction in BMI. This interaction may have promoted more conscious decision-making regarding diet and physical activity. However, it is also possible that the observed effects on BMI were secondary to the components’ effects on dietary behaviors, leading to reduced caloric intake.

Finally, mindfulness (C5) was found to be effective in reducing stress, both individually and in combination with other components. Mindfulness-based stress reduction is an established approach for reducing psychological stress and can be effectively delivered either in person or online [97,98]. While there was no strong evidence of effects on QoL, motivation (C3), in combination with skills/know-how (C4), showed some evidence of improving QoL. Extant interventions for improving QoL generally target individuals with chronic health conditions and are typically administered as part of integrated care interventions [99]. This study suggests that QoL may potentially be improved in the general population using digital interventions, possibly as a result of increased perceived self-efficacy arising from improvements in the 4 main health behavior domains.

### Generalizability and Limitations

We used a pragmatic approach in this trial and kept barriers to participation low. This strengthens the interpretation of the estimated effects as measures of effectiveness rather than efficacy. However, this comes with a trade-off with respect to attrition. Participants may have enrolled out of curiosity, and dropping out was easy; thus, attrition was, as expected, high. We found notable associations between nonresponse and age, as well as baseline smoking and more frequent episodes of heavy drinking. However, none of these associations were moderated by component access. Furthermore, although the evidence for these associations was relatively strong, the ORs were small. Thus, while the most conservative estimates of effects may be more relevant for older individuals, less frequent heavy drinkers, and smokers, overall, the evidence for systematic attrition with respect to responders versus nonresponders was not sufficiently strong to raise major concern. By contrast, there was evidence that late responders had higher fruit and vegetable consumption at follow-up compared with early responders, which, under the assumption that late responders and nonresponders are similar, suggests a risk of attrition bias. However, this association was not observed among those with access to the screening/feedback component (C1), which showed the most promising effects on fruit and vegetable consumption. Thus, the risk of bias for this finding in particular may be considered lower. Additionally, late responders had more MVPA minutes and were less likely to have quit smoking than early responders. Overall, this suggests that our estimates for these outcomes may be biased due to attrition; however, we do not have sufficient data to determine the direction or magnitude of this bias. Although this limitation should be considered when interpreting the findings, the primary aim of this study should also be emphasized: to examine the relative contributions of different intervention components to changes in health behaviors. The overall conclusions regarding which components are relatively more promising from an effectiveness perspective are less likely to be affected by attrition in this study than the precise effect estimates themselves.

The study used self-report measures to record outcomes. To minimize recall bias, we focused on behavior in the past week (except for heavy episodic drinking and smoking, which considered the past month). This avoided asking participants to recall behavior over several months or a year, which would not only be difficult to recall accurately but also less relevant given the time frame of the intervention. Although the validity of online measures has been established [100], they remain prone to social desirability or impression management biases [101]. However, in our study, blinding reduced the likelihood that these biases differed by allocation. For smoking and physical activity outcomes, intervention trials have used objective measures such as pedometers or salivary nicotine tests. Evidence suggests that self-reports may be less accurate than direct measures [102,103]; however, in this study, administering direct measures—particularly for smoking—was not feasible. Additionally, the study did not specify a particular type of physical activity; rather, participants reported minutes of activity. The Society for Research on Nicotine and Tobacco also recommends not using salivary tests in population studies to avoid introducing selection bias due to unequal opportunities to complete and return tests [68]. Conducting scientific studies requires decisions about which biases can be controlled and which must be accepted as limitations. In the case of self-report versus device-based measurement, there are trade-offs that are not uniform across studies. Rather than focusing solely on the accuracy of measured behavior—which is subject to bias regardless of method—future work should consider simulation studies to examine the impact of varying levels and types of bias. Estimating how measurement decisions influence effect estimates may help inform and justify study design choices.

An important consideration when assessing the generalizability of the findings from this factorial trial is the level of service utilization in a real-world implementation. If different groups in society are more or less likely to use digital interventions, the health benefits of implementation may disproportionately affect certain groups. Furthermore, the intervention materials may have differential effects across population subgroups, and not all groups may have been adequately represented in our study sample. Our estimates of effects are generalizable to the wider population insofar as it can be assumed that effects are not moderated by factors associated with nonparticipation. However, it is not possible to assess such moderation in this study, and dedicated studies are needed to examine implementation and moderation effects in greater detail.

Finally, this study targeted individuals who had sought help online; thus, participants were expected to have some motivation to change at study entry. This was supported by the high importance reported at baseline. Providing support to individuals actively seeking help is important; however, engaging those who could benefit from behavior change but are not actively seeking support remains a challenge. Future research should focus on proactive approaches to community outreach for multiple behavior support, potentially drawing on brief alcohol intervention screening strategies used in higher education settings [39,40]. This may include offering digital interventions to all individuals visiting primary health care clinics or other central community locations, such as pharmacies, grocery stores, and sports arenas. Relatedly, maintaining motivation throughout the behavior change process is critical for promoting long-term, sustained change. In this study, we provided motivational and tip-based reminders throughout the week; however, the optimal approach to sustaining motivation in digital interventions remains unclear. Strategies such as gamification [104] or habit formation [105] may be effective for some individuals. Future research should explore heterogeneity in sustained motivation and behavior change to better understand what drives long-term success.

### Conclusion

This study used a factorial design to estimate the effects of a digital multiple health behavior change intervention at the component level. Studying effects at the component level provided novel insights into the building blocks of complex digital health behavior change interventions. In addition, these effects were examined across multiple behaviors. Overall, the screening/feedback component was the most effective for changing health behaviors, along with goal-setting/planning and motivation to change. Several components interacted to further improve health outcomes, most notably screening/feedback with motivation to change, screening/feedback with skills/know-how, and motivation to change with skills/know-how. By contrast, some components also had detrimental effects on certain behaviors, suggesting that not all components are appropriate for all behaviors. The results from this study contribute to the development of more effective interventions by identifying which components of complex interventions are most effective for specific behaviors.

## Appendix

Multimedia Appendix 1 CONSORT-eHEALTH checklist (V 1.6.1).

Multimedia Appendix 2 Informed consent materials.

Multimedia Appendix 3 Questionnaires.

Multimedia Appendix 4 Intervention description and factorial conditions.

Multimedia Appendix 5 Attrition nonresponse analyses.

Multimedia Appendix 6 Attrition attempts analyses.

Multimedia Appendix 7 Estimates of effects (available data).

Multimedia Appendix 8 Estimates of effects (imputed data).

Multimedia Appendix 9 Estimates of effects of number of components.

## Abbreviations

CoI: compatibility interval
COM-B: Capability-Opportunity-Motivation-Behavior
CONSORT: Consolidated Standards of Reporting Trials
DALY: disability-adjusted life year
IRR: incidence rate ratio
MoBILE: Mobile Health Multiple Lifestyle Behavior Interventions Across the Lifespan
MVPA: moderate-to-vigorous physical activity
OR: odds ratio
POE: probability of effect
PROMIS: Patient-Reported Outcomes Measurement Information System
QoL: quality of life

## Data Availability

The datasets used or analyzed during this study are available from the corresponding author (MB) on reasonable request. Ethical approval is required for data sharing.
