# Supplementary material for: Effectiveness of the Components of a Digital Multiple Health Behavior Change Intervention Among Individuals Seeking Help Online (Coach): Factorial Randomized Trial
Source: J Med Internet Res. 2026 Apr 2;28:e88881. doi: 10.2196/88881 (PMC13087559; doi:10.2196/88881)
Supplement: Multimedia Appendix 2 [file jmir_v28i1e88881_app2.pdf]

MULTIMEDIA APPENDIX 2 – INFORMED CONSENT MATERIALS

We would like to ask you to participate in a research project. On this page you can find information about the project and what participation entails.

WHY ARE WE DOING THIS STUDY AND WHY DO YOU WANT ME TO PARTICIPATE?

The Coach-study is a research project which aims to evaluate the effects of a digital support tool for improved health behaviours. The support tool, which we call *Coach*, is delivered entirely through your mobile phone. We are asking you if you want to participate as you have shown interest in the study by sending us a text message after having searched online or visited a website where information about this study was presented.

The research project is conducted at Linköping University in Sweden, please find contact details for the primary investigator below.

WHAT IS EXPECTED OF ME?

Those who consent to take part in the study will then be asked to complete a short questionnaire about their current lifestyle behaviours. As Coach is designed to help participants change their alcohol, physical activity, diet and smoking behaviours, the questionnaire will contain questions about these. After the questionnaire has been completed, those who have at least one unhealthy lifestyle behaviour will be given access to Coach for four months. A text will be sent to participants immediately after the survey with information about how Coach works. As Coach is delivered through text messages, participants will not have to download any apps or do anything else than respond to the questionnaire to get going.

Coach consists of a text message that is sent to participants every Sunday for four months. In the text there will be a link taking participants to the support tool. There are different versions of Coach and chance alone decides which one is assigned to each participant. The materials will therefore be different for different participants, but overall, the support is focused on feedback and reflection on current behaviours with tips and tools to build strategies for change. The support may also include tools to build mental and physical resources for change, and recommendations to other available support. It is up to participants to decide to which degree the various tools are used, and the tools will be available for the full four months.

All participants will be asked to complete questionnaires regarding their current lifestyle behaviours one, two, and four months after Coach has started. The questionnaires take 5-10 minutes to complete. Participation is complete after four months.

WILL I BE TAKING ANY RISKS BY PARTICIPATING IN THIS STUDY?

If you decide to take part in the study then you should be aware that while Coach has been designed based on current scientific evidence regarding how to support individuals to change their behaviours, not everyone who uses Coach will succeed. This may feel like a failure and may be de-motivating. Participants should also be aware that changing lifestyle behaviours may result in discomfort, for instance withdrawal symptoms from alcohol or nicotine. These discomforts are passing, and in the long run the health benefits outweigh these discomforts.

You can always decide to stop Coach and we will not ask you why. Information about how to stop Coach will be given to you if you decide to participate. If you want to talk to somebody that is independent from the project you can contact REDACTED. You can also contact your primary healthcare centre or 1177 if you feel that you want more help with your health, or if you feel any discomforts.

WILL I BE LEAVING ANY PERSONAL INFORMATION?

The project will collect information about you.

Your responses to the questionnaires during the study period will be stored in a database at Linköping university. We will use an encrypted version of your phone number in order to connect your responses from the different questionnaires. The secret key used to decrypt the phone number will only be accessible by the primary investigator Marcus Bendtsen (see contact information below). We will also collect information about how you use Coach, so that we can analyse usage of the support tool.

When the project is complete, all phone numbers will be deleted, and the collected data will then become anonymous. The anonymous information will be stored securely at Linköpings University for 10 years.

The data collection is for scientific research and is therefore motivated by public interest (GDPR EU 2016/679, Prop. 2017/18:298).

Your data will be stored so that only authorised researchers have access to them. Linköping University are responsible for your data. According to EU:s data protection regulation you have the right to, free of charge, be given access to the data collected about you in the project, and have errors corrected. You may also request that your data be deleted or use of your data be restricted. If you want to be given access to your data, you should contact the primary investigator Marcus Bendtsen (see contact information below). The data protection officer can be reached at dataskyddsombud@liu.se. If you are not satisfied with the way your personal data has been handled, you can file a complaint at the Swedish Data Protection Authority.

HOW CAN I GET MORE INFORMATION ABOUT THE RESULTS FROM THIS STUDY?

After the study has been completed, findings will be published in scientific peer-reviewed journals. Findings at the individual level will not be traceable from these publications. We will not contact you after study completion, but you are welcome to contact us if you would like copies of published reports.

INSURENCE

As a participant of a research project at Linköping University you are included in the insurance the university has at Kammarkollegiet.

PARTICIPATION IS FREE

Participation is free and you can at any time decide to end it. If you decide to end your participation you will not be asked why, and it will not affect future care, treatment or your studies. If you wish to end your participation you should contact the primary investigator Marcus Bendtsen (see below).

HOW WILL THE SUPPORT BE DISSEMINATED?

If this project finds that Coach has a positive effect on health behaviours then it will be made freely available. The primary investigator Marcus Bendtsen owns a company (Alexit AB) which may be responsible for the dissemination. Alexit AB does not have access to any data from this study and has no influence on the research questions or analyses.
