# Supplementary material for: Effectiveness of the Components of a Digital Multiple Health Behavior Change Intervention Among Individuals Seeking Help Online (Coach): Factorial Randomized Trial
Source: J Med Internet Res. 2026 Apr 2;28:e88881. doi: 10.2196/88881 (PMC13087559; doi:10.2196/88881)
Supplement: Multimedia Appendix 3 [file jmir_v28i1e88881_app3.pdf]

MULTIMEDIA APPENDIX 3 – QUESTIONNAIRES

Note: Participants are reminded of the definition of a standard drink of alcohol by graphical means, as well as given visual cues for what constitutes a portion of fruit and vegetables.

BASELINE QUESTIONNAIRE

1. Sex:
   a. Female
   b. Male

2. Age (numerical measure)

3. How many standard drinks of alcohol did you consume last week? (numerical measure)

4. How often, during the past month, have you consumed four/five (female/male) or more standard drinks of alcohol on one occasion? (numerical measure)

5. How many cigarettes did you smoke last week? (numerical measure)

6. How much time in total did you spend on moderate physical activity (e.g. bicycling or walking for transport or leisure) **last week**?
   a. 0
   b. Less than 30 minutes
   c. 30-60 minutes
   d. 1 hours
   e. 1.5 hours
   f. 2 hours
   g. 2.5 hours
   h. 3 hours
   i. 3.5 hours (i.e. 30 minutes per day)
   j. 4 hours
   k. 5 hours
   l. 6 hours
   m. 7 hours (i.e. 1 hour per day)
   n. 10.5 hours (i.e. 1.5 hours per day)
   o. 14 hours (i.e. 2 hours per day)

7. How much time in total did you spend on vigorous physical activity (i.e. producing increases in breathing or heart rate), for instance running, aerobics, etc. **last week?**
   a. 0
   b. Less than 30 minutes
   c. 30-60 minutes
   d. 1 hours
   e. 1.5 hours
   f. 2 hours
   g. 2.5 hours
   h. 3 hours
   i. 3.5 hours (i.e. 30 minutes per day)

j. 4 hours
k. 5 hours
l. 6 hours
m. 7 hours (i.e. 1 hour per day)
n. 10.5 hours (i.e. 1.5 hours per day)
o. 14 hours (i.e. 2 hours per day)

8. How many 100g portions (equivalent to an average sized banana or one large apple) of fruit did you consume **last week**?
    a. 0
    b. 1-2 portions **per week**
    c. 3-4 portions **per week**
    d. 5-6 portion **per week**
    e. 1.0 portion **per day**
    f. 1.5 portions **per day**
    g. 2.0 portions **per day**
    h. 2.5 portions **per day**
    i. 3.0 portions **per day or more**

9. How many 100 g portions (equivalent to an average handful) of vegetables did you consume **last week**?
    a. 0
    b. 1-2 portions **per week**
    c. 3-4 portions **per week**
    d. 5-6 portion **per week**
    e. 1.0 portion **per day**
    f. 1.5 portions **per day**
    g. 2.0 portions **per day**
    h. 2.5 portions **per day**
    i. 3.0 portions **per day or more**

10. In the last month, how often have you felt that you were unable to control the important things in your life?
    a. Never
    b. Almost never
    c. Sometimes
    d. Fairly often
    e. Very often

11. In the last month, how often have you felt confident about your ability to handle your personal problems?
    a. Never
    b. Almost never
    c. Sometimes
    d. Fairly often
    e. Very often

12. In the last month, how often have you felt that things were going your way?
    a. Never
    b. Almost never

   c. Sometimes
   d. Fairly often
   e. Very often

13. In the last month, how often have you felt difficulties were piling up so high that you could not overcome them?
    a. Never
    b. Almost never
    c. Sometimes
    d. Fairly often
    e. Very often

14. How important is it for you to improve your lifestyle behaviours? (10-point scale ranging from 1 = “Not important” to 10 = “Very important”)

15. How confident are you that you will be able to improve your lifestyle behaviours? (10-point scale ranging from 1 = “Not at all” to 10 = “Very confident”)

16. To what degree do you have the know-how and strategies to improve your lifestyle behaviours? (10-point scale ranging from 1 = “Not at all” to 10 = “Very high degree”)

1-MONTH FOLLOW-UP (MEDIATORS ONLY) QUESTIONNAIRE

1. How important is it for you to improve your lifestyle behaviours? (10-point scale ranging from 1 = “Not important” to 10 = “Very important”)

2. How confident are you that you will be able to improve your lifestyle behaviours? (10-point scale ranging from 1 = “Not at all” to 10 = “Very confident”)

3. To what degree do you have the know-how and strategies to improve your lifestyle behaviours? (10-point scale ranging from 1 = “Not at all” to 10 = “Very high degree”)

2- AND 4-MONTH FOLLOW-UP QUESTIONNAIRE

1. How many standard drinks of alcohol did you consume last week? (numerical measure)

2. How often, during the past month, have you consumed four/five (female/male) or more standard drinks of alcohol on one occasion? (numerical measure)

3. Have you smoked any cigarettes the past four weeks?
   a. Yes
   b. No

4. (Smokers only) How many cigarettes did you smoke last week? (numerical measure)

5. How much time in total did you spend on moderate physical activity (e.g. bicycling or walking for transport or leisure) **last week**?
   a. 0
   b. Less than 30 minutes
   c. 30-60 minutes
   d. 1 hours

e. 1.5 hours
f. 2 hours
g. 2.5 hours
h. 3 hours
i. 3.5 hours (i.e. 30 minutes per day)
j. 4 hours
k. 5 hours
l. 6 hours
m. 7 hours (i.e. 1 hour per day)
n. 10.5 hours (i.e. 1.5 hours per day)
o. 14 hours (i.e. 2 hours per day)

6. How much time in total did you spend on vigorous physical activity (i.e. producing increases in breathing or heart rate), for instance running, aerobics, etc. **last week?**
    a. 0
    b. Less than 30 minutes
    c. 30-60 minutes
    d. 1 hours
    e. 1.5 hours
    f. 2 hours
    g. 2.5 hours
    h. 3 hours
    i. 3.5 hours (i.e. 30 minutes per day)
    j. 4 hours
    k. 5 hours
    l. 6 hours
    m. 7 hours (i.e. 1 hour per day)
    n. 10.5 hours (i.e. 1.5 hours per day)
    o. 14 hours (i.e. 2 hours per day)

7. How many 100g portions (equivalent to an average sized banana or one large apple) of fruit did you consume **last week**?
    a. 0
    b. 1-2 portions **per week**
    c. 3-4 portions **per week**
    d. 5-6 portion **per week**
    e. 1.0 portion **per day**
    f. 1.5 portions **per day**
    g. 2.0 portions **per day**
    h. 2.5 portions **per day**
    i. 3.0 portions **per day or more**

8. How many 100 g portions (equivalent to an average handful) of vegetables did you consume **last week**?
    a. 0
    b. 1-2 portions **per week**
    c. 3-4 portions **per week**
    d. 5-6 portion **per week**
    e. 1.0 portion **per day**
    f. 1.5 portions **per day**

g. 2.0 portions **per day**
h. 2.5 portions **per day**
i. 3.0 portions **per day or more**

9. How many cans (33 cl, one standard can) of sugary drinks (e.g. soft/fizzy drinks, “energy drinks”) did you consume **last week**?
    a. 0 cans
    b. 1 can **per week**
    c. 2-3 cans **per week**
    d. 4-6 cans **per week**
    e. 1 can **per day**
    f. 1.5 cans **per day**
    g. 2.0 cans **per day**
    h. 2.5 cans **per day**
    i. 3.0 cans **per day or more**

10. How many portions of sweets, chocolate, pastry (e.g. buns, muffins, biscuits), ice cream and salty snacks (e.g. crisps, nuts, cheese doodles) did you eat **last week**? *One portion is 50 g sweets (9 pieces), 40 g chocolate (6 pieces/squares), 1 bun, 2 dl (scoops) of ice cream or 2 dl snacks (40 g)*.

    a. 0 portions
    b. 1 portion **per week**
    c. 2-3 portions **per week**
    d. 4-6 portions **per week**
    e. 1 portion **per day**
    f. 1.5 portions **per day**
    g. 2.0 portions **per day**
    h. 2.5 portions **per day**
    i. 3.0 portions **per day**
    j. 3.5 portions **per day**
    k. 4.0 portions **per day or more**

11. How tall are you? (numerical measure)

12. What is your current body weight? (numerical measure)

13. In the last month, how often have you felt that you were unable to control the important things in your life?
    a. Never
    b. Almost never
    c. Sometimes
    d. Fairly often
    e. Very often

14. In the last month, how often have you felt confident about your ability to handle your personal problems?
    a. Never
    b. Almost never

    c. Sometimes
    d. Fairly often
    e. Very often

15. In the last month, how often have you felt that things were going your way?
    a. Never
    b. Almost never
    c. Sometimes
    d. Fairly often
    e. Very often

16. In the last month, how often have you felt difficulties were piling up so high that you could not overcome them?
    a. Never
    b. Almost never
    c. Sometimes
    d. Fairly often
    e. Very often

17. How important is it for you to improve or maintain healthy lifestyle behaviours? (10-point scale ranging from 1 = "Not important" to 10 = "Very important")

18. How confident are you that you will be able to improve or maintain healthy lifestyle behaviours? (10-point scale ranging from 1 = "Not at all" to 10 = "Very confident")

19. To what degree do you have the know-how and strategies to improve or maintain healthy lifestyle behaviours? (10-point scale ranging from 1 = "Not at all" to 10 = "Very high degree")

4-MONTH FOLLOW-UP ONLY

1. Overall, how well do you believe that the support given to you suited your needs?
    a. I feel like I did not receive any support at all
    b. I feel like I received some support, but it did not suit my needs
    c. I feel like I received some support, and it did suit my needs
    d. I feel like I received all the support that I needed

2. (If a or b to question 2): You have responded that you did not receive adequate support, what did you do instead?
    a. I decided to find other ways to help me change my lifestyle
    b. I decided to not make any change to my lifestyle
    c. Other (please comment)

3. Please leave a comment describing your needs and how the support did or did not address them (Free-text).

4. Do you believe that the support given to you would be helpful for other individuals that want to change their lifestyle? (1 = "Not very helpful" to 5 = "Very helpful")

5. Would you recommend the support you were given to a friend who expresses a wish to change their lifestyle?

    a. Yes
    b. No
    c. I do not know

6. If you were to continue using the support, for how much longer would you want to use it?
    a. I would use it for one to two more months
    b. I would use it for three to six more months
    c. I would use it for more than six months
    d. I would not use it any more
    e. I do not know

7. In general, would you say your health is: (Poor, Fair, Good, Very good, Excellent)

8. In general, would you say your quality of life is: (Poor, Fair, Good, Very good, Excellent)

9. In general, how would you rate your physical health: (Poor, Fair, Good, Very good, Excellent)

10. In general, how would you rate your mental health, including your mood and your ability to think? (Poor, Fair, Good, Very good, Excellent)

11. In general, how would you rate your satisfaction with your social activities and relationships? (Poor, Fair, Good, Very good, Excellent)

12. In general, please rate how well you carry out your usual social activities. This includes activities at home, at work and in your community, and responsibilities as a parent, child, spouse, employee, friend, etc.: (Poor, Fair, Good, Very good, Excellent)

13. To what extent are you able to carry out your everyday physical activities such as walking, climbing stairs, carrying groceries, or moving a chair?
    a. Not at all
    b. A little
    c. Moderately
    d. Mostly
    e. Completely

14. In the past 7 days, how often have you been bothered by emotional problems such as feeling anxious depressed or irritable?
    a. Always
    b. Often
    c. Sometimes
    d. Rarely
    e. Never

15. In the past 7 days, how would you rate your fatigue on average?
    a. Very severe
    b. Severe
    c. Moderate
    d. Mild
    e. None

16. In the past 7 days, how would you rate your pain on average (where 0 is No Pain, and 10 is Worst Pain Imaginable)?
