# Supplementary material for: Effectiveness of the Components of a Digital Multiple Health Behavior Change Intervention Among Individuals Seeking Help Online (Coach): Factorial Randomized Trial
Source: J Med Internet Res. 2026 Apr 2;28:e88881. doi: 10.2196/88881 (PMC13087559; doi:10.2196/88881)
Supplement: Multimedia Appendix 5 [file jmir_v28i1e88881_app5.pdf]

MULTIMEDIA APPENDIX 5 – ATTRITION NON-RESPONSE ANALYSES

Component abbreviations:

C1 = Screening and feedback
C2 = Goal-setting and planning
C3 = Motivation
C4 = Skills and know-how
C5 = Mindfulness
C6 = Self-authored text messages

Statistical analysis:

We studied attrition by estimating the odds of responding to follow-up conditional on baseline characteristics and presence or absence of the six components (factors) using logistic regression. We estimated one model with no interaction terms and a second model where we included interaction terms between factor and baseline characteristics. We used Cauchy priors to promote a parsimonious model (cantered at 0 with a standard normal hyper prior for scale).

SUMMARY

Estimates of association between baseline characteristics and non-response at the 2-month follow-up interval are presented in Supplementary Table D1, and with interaction terms with components in Supplementary Table D2. Likewise, estimates of associations between baseline characteristics and non-response at the 4-month follow-up interval are presented in Supplementary Table D3 and Supplementary Table D4.

Overall, we found evidence that younger participants were less likely to respond to follow-up than older participants at both follow-up intervals. In addition, those with more frequent episodes of heavy drinking and who smoked more at baseline were also less likely to respond to follow-up. Notably, these associations were not moderated by differential access to components.

TABLES

Supplementary Table D1 - Associations between baseline characterstics and non-response to the 2-month follow-up

	Est.	Prob.
C1	1.01 (0.99; 1.18)	75.2%
C2	1.00 (0.96; 1.04)	52.6%
C3	1.00 (0.98; 1.09)	63.9%
C4	1.00 (0.91; 1.02)	63.4%
C5	1.00 (0.98; 1.07)	59.6%
C6	1.00 (0.94; 1.03)	56.4%
Man vs. Woman	0.99 (0.80; 1.02)	73.7%
Age	0.98 (0.98; 0.99)	> 99.9%
Heavy episodic drinking	1.02 (1.01; 1.04)	> 99.9%
Weekly alcohol consumption	1.00 (1.00; 1.01)	82.5%
Weekly moderate and vigorous physical activity	1.00 (1.00; 1.00)	81.3%
Average daily portions of fruit and vegetables	1.00 (0.96; 1.02)	62.4%
Number of cigarettes smoked per week	1.01 (1.00; 1.01)	> 99.9%
Perceived stress	1.01 (1.00; 1.04)	90.5%
Importance of change	1.00 (0.97; 1.02)	56.8%

Confidence in ability to change	1.00 (0.98; 1.01)	58.9%
Knowledge of how to change	1.00 (0.98; 1.01)	63.0%

Est. – Median of the posterior distribution of odds ratios with 95% compatibility intervals.
Prob. – Proportion of the posterior distribution above or below the null in the direction of the median.

Supplementary Table D2 - Associations between baseline characterstics and non-response to the 2-month follow-up with interactions between characteristics and intervention components

	Est.	Prob.
C1	1.00 (1.00; 1.00)	50.7%
C2	1.00 (1.00; 1.00)	50.3%
C3	1.00 (1.00; 1.00)	50.4%
C4	1.00 (1.00; 1.00)	50.0%
C5	1.00 (1.00; 1.00)	50.0%
C6	1.00 (1.00; 1.00)	50.3%
Man vs. Woman	1.00 (0.99; 1.00)	51.6%
Age	0.98 (0.98; 0.99)	> 99.9%
Heavy episodic drinking	1.03 (1.02; 1.04)	99.9%
Weekly alcohol consumption	1.00 (1.00; 1.00)	55.5%
Weekly moderate and vigorous physical activity	1.00 (1.00; 1.00)	68.8%
Average daily portions of fruit and vegetables	1.00 (1.00; 1.00)	51.7%
Number of cigarettes smoked per week	1.00 (1.00; 1.01)	> 99.9%
Perceived stress	1.00 (1.00; 1.02)	57.1%
Importance of change	1.00 (1.00; 1.00)	50.5%
Confidence in ability to change	1.00 (1.00; 1.00)	51.5%
Knowledge of how to change	1.00 (1.00; 1.00)	52.1%
**C1 interactions**		
C1 x Man vs. Woman	1.00 (1.00; 1.00)	50.6%
C1 x Age	1.00 (1.00; 1.00)	70.0%
C1 x Heavy episodic drinking	1.00 (1.00; 1.00)	51.8%
C1 x Weekly alcohol consumption	1.00 (1.00; 1.00)	52.6%
C1 x Weekly moderate and vigorous physical activity	1.00 (1.00; 1.00)	85.7%
C1 x Average daily portions of fruit and vegetables	1.00 (1.00; 1.00)	50.6%
C1 x Number of cigarettes smoked per week	1.00 (1.00; 1.00)	57.1%
C1 x Perceived stress	1.00 (1.00; 1.01)	57.7%
C1 x Importance of change	1.00 (1.00; 1.01)	55.5%
C1 x Confidence in ability to change	1.00 (1.00; 1.00)	51.9%
C1 x Knowledge of how to change	1.00 (1.00; 1.00)	52.3%
**C2 interactions**		
C2 x Man vs. Woman	1.00 (1.00; 1.00)	50.7%
C2 x Age	1.00 (1.00; 1.00)	55.3%
C2 x Heavy episodic drinking	1.00 (1.00; 1.00)	52.1%
C2 x Weekly alcohol consumption	1.00 (1.00; 1.00)	52.9%
C2 x Weekly moderate and vigorous physical activity	1.00 (1.00; 1.00)	56.8%
C2 x Average daily portions of fruit and vegetables	1.00 (1.00; 1.00)	51.0%
C2 x Number of cigarettes smoked per week	1.00 (1.00; 1.00)	68.6%

C2 x Perceived stress	1.00 (1.00; 1.00)	52.0%
C2 x Importance of change	1.00 (1.00; 1.00)	50.3%
C2 x Confidence in ability to change	1.00 (1.00; 1.00)	52.8%
C2 x Knowledge of how to change	1.00 (1.00; 1.00)	52.3%
**C3 interactions**		
C3 x Man vs. Woman	1.00 (1.00; 1.00)	51.3%
C3 x Age	1.00 (1.00; 1.00)	64.3%
C3 x Heavy episodic drinking	1.00 (1.00; 1.00)	50.2%
C3 x Weekly alcohol consumption	1.00 (1.00; 1.00)	50.0%
C3 x Weekly moderate and vigorous physical activity	1.00 (1.00; 1.00)	61.9%
C3 x Average daily portions of fruit and vegetables	1.00 (1.00; 1.00)	50.2%
C3 x Number of cigarettes smoked per week	1.00 (1.00; 1.00)	63.1%
C3 x Perceived stress	1.00 (1.00; 1.01)	57.7%
C3 x Importance of change	1.00 (1.00; 1.00)	51.6%
C3 x Confidence in ability to change	1.00 (1.00; 1.00)	50.5%
C3 x Knowledge of how to change	1.00 (1.00; 1.00)	50.7%
**C4 interactions**		
C4 x Man vs. Woman	1.00 (1.00; 1.00)	50.1%
C4 x Age	1.00 (1.00; 1.00)	55.3%
C4 x Heavy episodic drinking	1.00 (1.00; 1.00)	51.1%
C4 x Weekly alcohol consumption	1.00 (1.00; 1.00)	55.0%
C4 x Weekly moderate and vigorous physical activity	1.00 (1.00; 1.00)	93.3%
C4 x Average daily portions of fruit and vegetables	1.00 (1.00; 1.00)	51.6%
C4 x Number of cigarettes smoked per week	1.00 (1.00; 1.00)	53.4%
C4 x Perceived stress	1.00 (1.00; 1.00)	53.6%
C4 x Importance of change	1.00 (1.00; 1.00)	50.6%
C4 x Confidence in ability to change	1.00 (1.00; 1.00)	52.3%
C4 x Knowledge of how to change	1.00 (1.00; 1.00)	54.2%
**C5 interactions**		
C5 x Man vs. Woman	1.00 (1.00; 1.00)	51.1%
C5 x Age	1.00 (1.00; 1.00)	56.5%
C5 x Heavy episodic drinking	1.00 (1.00; 1.00)	50.7%
C5 x Weekly alcohol consumption	1.00 (1.00; 1.00)	56.9%
C5 x Weekly moderate and vigorous physical activity	1.00 (1.00; 1.00)	82.8%
C5 x Average daily portions of fruit and vegetables	1.00 (1.00; 1.00)	51.2%
C5 x Number of cigarettes smoked per week	1.00 (1.00; 1.00)	52.8%
C5 x Perceived stress	1.00 (1.00; 1.00)	54.4%
C5 x Importance of change	1.00 (1.00; 1.00)	52.7%
C5 x Confidence in ability to change	1.00 (1.00; 1.00)	50.5%
C5 x Knowledge of how to change	1.00 (1.00; 1.00)	51.2%
**C6 interactions**		
C6 x Man vs. Woman	1.00 (1.00; 1.00)	50.7%
C6 x Age	1.00 (1.00; 1.00)	52.6%
C6 x Heavy episodic drinking	1.00 (1.00; 1.00)	50.1%
C6 x Weekly alcohol consumption	1.00 (1.00; 1.00)	53.3%
C6 x Weekly moderate and vigorous physical activity	1.00 (1.00; 1.00)	79.9%

C6 x Average daily portions of fruit and vegetables	1.00 (1.00; 1.00)	50.4%
C6 x Number of cigarettes smoked per week	1.00 (1.00; 1.00)	52.8%
C6 x Perceived stress	1.00 (1.00; 1.00)	51.1%
C6 x Importance of change	1.00 (1.00; 1.00)	51.0%
C6 x Confidence in ability to change	1.00 (1.00; 1.00)	51.4%
C6 x Knowledge of how to change	1.00 (1.00; 1.00)	50.1%

Est. – Median of the posterior distribution of odds ratios with 95% compatibility intervals.
Prob. – Proportion of the posterior distribution above or below the null in the direction of the median.

Supplementary Table D3 - Associations between baseline characterstics and non-response to the 4-month follow-up

	Est.	Prob.
C1	1.00 (0.98; 1.12)	68.7%
C2	1.00 (0.95; 1.04)	52.6%
C3	1.00 (0.97; 1.05)	53.2%
C4	1.00 (0.95; 1.03)	55.2%
C5	1.00 (0.96; 1.04)	50.8%
C6	1.00 (0.92; 1.02)	62.9%
Man vs. Woman	0.98 (0.77; 1.01)	79.7%
Age	0.99 (0.98; 0.99)	> 99.9%
Heavy episodic drinking	1.03 (1.02; 1.04)	> 99.9%
Weekly alcohol consumption	1.01 (1.00; 1.02)	87.0%
Weekly moderate and vigorous physical activity	1.00 (1.00; 1.00)	72.4%
Average daily portions of fruit and vegetables	1.00 (0.95; 1.01)	69.2%
Number of cigarettes smoked per week	1.01 (1.00; 1.01)	> 99.9%
Perceived stress	1.01 (1.00; 1.03)	87.8%
Importance of change	1.00 (0.99; 1.04)	71.9%
Confidence in ability to change	1.00 (0.99; 1.02)	67.3%
Knowledge of how to change	1.00 (0.98; 1.02)	50.3%

Est. – Median of the posterior distribution of odds ratios with 95% compatibility intervals.
Prob. – Proportion of the posterior distribution above or below the null in the direction of the median.

Supplementary Table D4 - Associations between baseline characterstics and non-response to the 4-month follow-up with interactions between characteristics and intervention components

	Est.	Prob.
C1	1.00 (1.00; 1.00)	50.3%
C2	1.00 (1.00; 1.00)	50.1%
C3	1.00 (1.00; 1.00)	50.9%
C4	1.00 (1.00; 1.00)	50.2%
C5	1.00 (1.00; 1.00)	50.2%
C6	1.00 (1.00; 1.00)	50.3%
Man vs. Woman	1.00 (0.95; 1.00)	53.1%
Age	0.99 (0.98; 1.00)	99.9%
Heavy episodic drinking	1.03 (1.02; 1.04)	> 99.9%

Weekly alcohol consumption	1.00 (1.00; 1.01)	56.2%
Weekly moderate and vigorous physical activity	1.00 (1.00; 1.00)	56.2%
Average daily portions of fruit and vegetables	1.00 (1.00; 1.00)	51.8%
Number of cigarettes smoked per week	1.01 (1.00; 1.01)	99.9%
Perceived stress	1.00 (1.00; 1.01)	56.8%
Importance of change	1.00 (1.00; 1.01)	53.2%
Confidence in ability to change	1.00 (1.00; 1.00)	51.6%
Knowledge of how to change	1.00 (1.00; 1.00)	50.1%
**C1 interactions**		
C1 x Man vs. Woman	1.00 (1.00; 1.00)	51.1%
C1 x Age	1.00 (1.00; 1.00)	59.1%
C1 x Heavy episodic drinking	1.00 (1.00; 1.00)	51.6%
C1 x Weekly alcohol consumption	1.00 (1.00; 1.00)	55.9%
C1 x Weekly moderate and vigorous physical activity	1.00 (1.00; 1.00)	94.5%
C1 x Average daily portions of fruit and vegetables	1.00 (1.00; 1.00)	50.5%
C1 x Number of cigarettes smoked per week	1.00 (1.00; 1.00)	53.7%
C1 x Perceived stress	1.00 (1.00; 1.00)	53.2%
C1 x Importance of change	1.00 (1.00; 1.00)	52.2%
C1 x Confidence in ability to change	1.00 (1.00; 1.00)	50.4%
C1 x Knowledge of how to change	1.00 (1.00; 1.01)	54.0%
**C2 interactions**		
C2 x Man vs. Woman	1.00 (0.99; 1.00)	51.7%
C2 x Age	1.00 (1.00; 1.00)	66.4%
C2 x Heavy episodic drinking	1.00 (1.00; 1.00)	51.4%
C2 x Weekly alcohol consumption	1.00 (1.00; 1.00)	52.7%
C2 x Weekly moderate and vigorous physical activity	1.00 (1.00; 1.00)	76.2%
C2 x Average daily portions of fruit and vegetables	1.00 (0.99; 1.00)	53.1%
C2 x Number of cigarettes smoked per week	1.00 (1.00; 1.00)	62.1%
C2 x Perceived stress	1.00 (1.00; 1.00)	52.0%
C2 x Importance of change	1.00 (1.00; 1.00)	50.9%
C2 x Confidence in ability to change	1.00 (1.00; 1.00)	53.3%
C2 x Knowledge of how to change	1.00 (0.99; 1.00)	57.7%
**C3 interactions**		
C3 x Man vs. Woman	1.00 (1.00; 1.00)	50.6%
C3 x Age	1.00 (1.00; 1.00)	50.1%
C3 x Heavy episodic drinking	1.00 (1.00; 1.00)	50.3%
C3 x Weekly alcohol consumption	1.00 (1.00; 1.00)	51.3%
C3 x Weekly moderate and vigorous physical activity	1.00 (1.00; 10.0)	61.9%
C3 x Average daily portions of fruit and vegetables	1.00 (1.00; 1.00)	50.2%
C3 x Number of cigarettes smoked per week	1.00 (1.00; 1.00)	73.6%
C3 x Perceived stress	1.00 (1.00; 1.01)	57.8%
C3 x Importance of change	1.00 (1.00; 1.00)	51.5%
C3 x Confidence in ability to change	1.00 (1.00; 1.00)	51.1%
C3 x Knowledge of how to change	1.00 (1.00; 1.00)	50.4%
**C4 interactions**		
C4 x Man vs. Woman	1.00 (1.00; 1.00)	50.7%

C4 x Age	1.00 (1.00; 1.00)	58.7%
C4 x Heavy episodic drinking	1.00 (1.00; 1.00)	50.4%
C4 x Weekly alcohol consumption	1.00 (1.00; 1.00)	53.9%
C4 x Weekly moderate and vigorous physical activity	1.00 (1.00; 1.00)	79.4%
C4 x Average daily portions of fruit and vegetables	1.00 (1.00; 1.00)	51.8%
C4 x Number of cigarettes smoked per week	1.00 (1.00; 1.00)	64.8%
C4 x Perceived stress	1.00 (1.00; 1.00)	54.8%
C4 x Importance of change	1.00 (1.00; 1.00)	52.0%
C4 x Confidence in ability to change	1.00 (1.00; 1.00)	51.1%
C4 x Knowledge of how to change	1.00 (1.00; 1.00)	51.9%
**C5 interactions**		
C5 x Man vs. Woman	1.00 (1.00; 1.00)	50.9%
C5 x Age	1.00 (1.00; 1.00)	52.0%
C5 x Heavy episodic drinking	1.00 (1.00; 1.00)	50.1%
C5 x Weekly alcohol consumption	1.00 (1.00; 1.00)	52.6%
C5 x Weekly moderate and vigorous physical activity	1.00 (1.00; 1.00)	77.1%
C5 x Average daily portions of fruit and vegetables	1.00 (1.00; 1.00)	50.9%
C5 x Number of cigarettes smoked per week	1.00 (1.00; 1.00)	56.0%
C5 x Perceived stress	1.00 (1.00; 1.01)	56.0%
C5 x Importance of change	1.00 (1.00; 1.01)	54.3%
C5 x Confidence in ability to change	1.00 (1.00; 1.00)	52.1%
C5 x Knowledge of how to change	1.00 (1.00; 1.00)	50.7%
**C6 interactions**		
C6 x Man vs. Woman	1.00 (1.00; 1.00)	51.6%
C6 x Age	1.00 (1.00; 1.00)	53.9%
C6 x Heavy episodic drinking	1.00 (1.00; 1.00)	50.5%
C6 x Weekly alcohol consumption	1.00 (1.00; 1.00)	53.7%
C6 x Weekly moderate and vigorous physical activity	1.00 (1.00; 1.00)	91.5%
C6 x Average daily portions of fruit and vegetables	1.00 (1.00; 1.00)	50.3%
C6 x Number of cigarettes smoked per week	1.00 (1.00; 1.00)	58.6%
C6 x Perceived stress	1.00 (1.00; 1.00)	51.3%
C6 x Importance of change	1.00 (1.00; 1.00)	50.3%
C6 x Confidence in ability to change	1.00 (1.00; 1.00)	51.7%
C6 x Knowledge of how to change	1.00 (1.00; 1.00)	51.1%

Est. – Median of the posterior distribution of odds ratios with 95% compatibility intervals.
Prob. – Proportion of the posterior distribution above or below the null in the direction of the median.
