# Supplementary material for: Effectiveness of the Components of a Digital Multiple Health Behavior Change Intervention Among Individuals Seeking Help Online (Coach): Factorial Randomized Trial
Source: J Med Internet Res. 2026 Apr 2;28:e88881. doi: 10.2196/88881 (PMC13087559; doi:10.2196/88881)
Supplement: Multimedia Appendix 6 [file jmir_v28i1e88881_app6.pdf]

MULTIMEDIA APPENDIX 6 – ATTRITION ATTEMPTS ANALYSES

Component abbreviations:
C1 = Screening and feedback
C2 = Goal-setting and planning
C3 = Motivation
C4 = Skills and know-how
C5 = Mindfulness
C6 = Self-authored text messages

Statistical analysis:
We modelled primary outcome data conditional on the number of attempts to collect follow-up with interaction terms with each component. All models were further adjusted for presence/absence of all components, as well as baseline measures of age, sex, importance, confidence, know-how, and each respective outcome measured at baseline.

SUMMARY

We found evidence that late responders had higher consumption of fruits and vegetables than early responders at the 2- and 4-month follow-up interval. However, this association was moderated downwards for participants with access to C1, suggesting that those with access to this component did not show this association. We also found evidence that late responders had higher levels of MVPA than early responders at the 4-month interval only, with the association attenuated down for those with access to C5 and up with those with access to C6. For smoking cessation, we found evidence that suggested that late responders were less likely to have stopped smoking compared to early responders, but not so for those with access to C6 at the 2-month interval and C2 and C3 at the 4-month interval.

Overall, we found evidence that suggests that late responders differed from early responders. The degree to which this is due to systematic attrition or due to chance is not possible to estimate, but under the assumption that late responders are more alike non-responders than early responders are, then this is evidence of systematic attrition.

TABLES

WEEKLY ALCOHOL CONSUMPTION

Supplementary Table E1 - Associations between weekly alcohol consumption and attempts to collect follow-up at the 2- and 4-month follow-up interval

	Est.	Prob.
**2-month follow-up**		
Attempts	1.05 (0.98; 1.12)	91.8%
C1 x Attempts	0.98 (0.93; 1.03)	78.8%
C2 x Attempts	1.00 (0.95; 1.05)	55.4%
C3 x Attempts	0.98 (0.93; 1.03)	82.2%
C4 x Attempts	0.97 (0.92; 1.02)	87.0%
C5 x Attempts	1.02 (0.97; 1.08)	79.7%
C6 x Attempts	0.98 (0.93; 1.03)	79.9%
**4-month follow-up**		
Attempts	1.06 (0.96; 1.17)	88.4%

C1 x Attempts	0.97 (0.91; 1.04)	81.0%
C2 x Attempts	1.00 (0.93; 1.07)	53.5%
C3 x Attempts	0.99 (0.93; 1.06)	58.4%
C4 x Attempts	0.99 (0.92; 1.06)	66.4%
C5 x Attempts	0.98 (0.92; 1.05)	71.9%
C6 x Attempts	0.99 (0.92; 1.06)	62.9%
**Est.** – Median of the posterior distribution of incidence rate ratios with 95% compatibility intervals.<br>**Prob.** – Proportion of the posterior distribution above or below the null in the direction of the median.		

HEAVY EPISODIC DRINKING

Supplementary Table E2 - Associations between monthly episodes of heavy drinking and attempts to collect follow-up at the 2- and 4-month follow-up interval

	Est.	Prob.
**2-month follow-up**		
Attempts	1.05 (0.99; 1.12)	92.6%
C1 x Attempts	0.99 (0.95; 1.04)	63.0%
C2 x Attempts	1.02 (0.97; 1.07)	73.9%
C3 x Attempts	0.98 (0.93; 1.03)	82.2%
C4 x Attempts	1.00 (0.96; 1.05)	53.6%
C5 x Attempts	0.99 (0.95; 1.04)	63.1%
C6 x Attempts	1.00 (0.95; 1.05)	58.1%
**4-month follow-up**		
Attempts	0.96 (0.89; 1.05)	80.1%
C1 x Attempts	1.01 (0.95; 1.07)	59.9%
C2 x Attempts	0.99 (0.94; 1.06)	58.0%
C3 x Attempts	1.01 (0.95; 1.07)	60.9%
C4 x Attempts	1.03 (0.98; 1.10)	86.6%
C5 x Attempts	1.05 (0.99; 1.12)	94.3%
C6 x Attempts	0.98 (0.93; 1.04)	72.7%
**Est.** – Median of the posterior distribution of incidence rate ratios with 95% compatibility intervals.<br>**Prob.** – Proportion of the posterior distribution above or below the null in the direction of the median.		

FRUIT AND VEGETABLES

Supplementary Table E3 - Associations between average daily portions of fruit and vegetables and attempts to collect follow-up at the 2- and 4-month follow-up intervals

	Est.	Prob.
**2-month follow-up**		
Attempts	0.04 (-0.01; 0.09)	93.4%
C1 x Attempts	-0.05 (-0.09; -0.01)	99.5%
C2 x Attempts	0.05 (0.01; 0.09)	99.4%
C3 x Attempts	0.00 (-0.04; 0.04)	50.6%
C4 x Attempts	-0.02 (-0.05; 0.02)	82.8%
C5 x Attempts	-0.02 (-0.05; 0.02)	79.8%
C6 x Attempts	-0.01 (-0.04; 0.03)	64.9%

**4-month follow-up**		
Attempts	0.11 (0.05; 0.17)	> 99.9%
C1 x Attempts	-0.06 (-0.10; -0.02)	99.6%
C2 x Attempts	0.0 (-0.05; 0.04)	57.7%
C3 x Attempts	-0.01 (-0.05; 0.04)	60.6%
C4 x Attempts	0.0 (-0.05; 0.04)	54.8%
C5 x Attempts	-0.01 (-0.05; 0.04)	63.0%
C6 x Attempts	0.02 (-0.02; 0.06)	80.0%

Est. – Median of the posterior distribution of linear associations with 95% compatibility intervals.
Prob. – Proportion of the posterior distribution above or below the null in the direction of the median.

MODERATE AND VIGOROUS PHYSICAL ACTIVITY

Supplementary Table E4 - Associations between weekly moderate and vigorous physical activity and attempts to collect follow-up at the 2- and 4-month follow-up interval

	**Est.**	**Prob.**
**2-month follow-up**		
Attempts	8.0 (-5.6; 21.6)	87.8%
C1 x Attempts	-6.3 (-16.1; 3.5)	89.6%
C2 x Attempts	-1.6 (-11.4; 8.2)	62.9%
C3 x Attempts	4.9 (-4.9; 14.5)	83.5%
C4 x Attempts	3.04 (-7.0; 12.9)	72.8%
C5 x Attempts	-1.12 (-10.9; 8.7)	58.9%
C6 x Attempts	6.04 (-3.8; 16.0)	88.7%
**4-month follow-up**		
Attempts	22.2 (7.5; 37.3)	99.9%
C1 x Attempts	-7.5 (-18.4; 3.2)	91.8%
C2 x Attempts	1.5 (-9.2; 12.1)	60.9%
C3 x Attempts	-7.7 (-18.4; 3.0)	92.2%
C4 x Attempts	5.4 (-5.2; 16.2)	84.1%
C5 x Attempts	-11.1 (-21.9; -0.33)	97.9%
C6 x Attempts	10.5 (-0.36; 21.4)	97.1%

Est. – Median of the posterior distribution of linear associations with 95% compatibility intervals.
Prob. – Proportion of the posterior distribution above or below the null in the direction of the median.

SMOKING CESSATION

Supplementary Table E5 - Associations between smoking cessation and attempts to collect follow-up at the 2- and 4-month follow-up intervals

	**Est.**	**Prob.**
**2-month follow-up**		
Attempts	0.73 (0.57; 0.93)	99.5%
C1 x Attempts	1.11 (0.92; 1.34)	85.4%
C2 x Attempts	0.94 (0.78; 1.14)	74.6%
C3 x Attempts	1.03 (0.85; 1.24)	61.6%
C4 x Attempts	1.10 (0.92; 1.33)	84.4%

C5 x Attempts	0.93 (0.76; 1.12)	77.6%
C6 x Attempts	1.28 (1.06; 1.55)	99.4%
**4-month follow-up**		
Attempts	0.62 (0.47; 0.83)	99.9%
C1 x Attempts	1.02 (0.83; 1.25)	56.5%
C2 x Attempts	1.24 (1.01; 1.54)	97.9%
C3 x Attempts	1.33 (1.09; 1.65)	99.7%
C4 x Attempts	0.99 (0.80; 1.22)	53.9%
C5 x Attempts	1.09 (0.90; 1.34)	80.4%
C6 x Attempts	0.93 (0.76; 1.155)	74.5%

Est. – Median of the posterior distribution of odds ratios with 95% compatibility intervals.

Prob. – Proportion of the posterior distribution above or below the null in the direction of the median.
