# Supplementary material for: Effectiveness of the Components of a Digital Multiple Health Behavior Change Intervention Among Individuals Seeking Help Online (Coach): Factorial Randomized Trial
Source: J Med Internet Res. 2026 Apr 2;28:e88881. doi: 10.2196/88881 (PMC13087559; doi:10.2196/88881)
Supplement: Multimedia Appendix 8 [file jmir_v28i1e88881_app8.pdf]

MULTIMEDIA APPENDIX 8 – ESTIMATES OF EFFECTS (IMPUTED DATA)

The tables in this appendix present the estimated effects of individual and pairwise combinations of components on primary and secondary outcome measures. The analyses were done using imputed data.

For primary outcomes, analyses were among those who were indicated as having unhealthy behaviors at baseline, e.g. those included in analyses of outcomes of moderate and vigorous physical activity were those who had less then 150 minutes at baseline.

The first part of each table shows estimates of effects for the individual components at each follow-up interval. The second part of the table, under the heading "two-way interactions", shows the estimated effects of pairwise combinations of components.

Component abbreviations:

C1 = Screening and feedback
C2 = Goal-setting and planning
C3 = Motivation
C4 = Skills and know-how
C5 = Mindfulness
C6 = Self-authored text messages

Statistical analysis:

We contrasted presence versus absence of components using multilevel regression models with covariates for time-by-component interactions and participant level adaptative intercepts. We also estimated models with pairwise interactions between components. All models were adjusted for presence/absence of all components, as well as baseline measures of age, sex, importance, confidence, and know-how. Primary outcomes, stress, and weekly number of cigarettes smoked were additionally adjusted for their respective measures at baseline. BMI, sugary drinks, and sweets and snacks were adjusted for baseline MVPA minutes per week and average intake of fruit and vegetables per day. QoL was adjusted for perceived stress at baseline.

Supplementary Table G1 - Estimates of effects of components, including two-way interactions, on total weekly alcohol consumption at 2- and 4-month follow-up (imputed data).

	C1		C2		C3		C4		C5		C6	
	**Est.**	**Prob.**	**Est.**	**Prob.**	**Est.**	**Prob.**	**Est.**	**Prob.**	**Est.**	**Prob.**	**Est.**	**Prob.**
**2-month**	1.016 (0.921; 1.121)	62.1%	0.952 (0.858; 1.052)	82.8%	1.012 (0.915; 1.121)	59.1%	1.018 (0.916; 1.127)	63.1%	1.018 (0.921; 1.127)	63.3%	1.038 (0.936; 1.151)	75.9%
**4-month**	0.949 (0.853; 1.055)	83.0%	0.979 (0.881; 1.094)	65.4%	1.026 (0.924; 1.139)	68.3%	0.979 (0.883; 1.093)	64.8%	1.015 (0.912; 1.13)	60.4%	1.013 (0.906; 1.13)	59.1%
**Two-way interactions**												
**C2**												
**2-month**	0.966 (0.834; 1.116)	67.7%										
**4-month**	0.93 (0.797; 1.082)	82.3%										
**C3**												
**2-month**	1.028 (0.895; 1.185)	65.1%	0.964 (0.838; 1.108)	69.7%								
**4-month**	0.973 (0.838; 1.137)	63.5%	1.005 (0.866; 1.166)	52.5%								
**C4**												
**2-month**	1.034 (0.899; 1.19)	67.8%	0.97 (0.839; 1.116)	66.5%	1.03 (0.892; 1.19)	65.4%						
**4-month**	0.929 (0.801; 1.084)	82.5%	0.957 (0.826; 1.124)	70.7%	1.005 (0.865; 1.171)	52.5%						
**C5**												
**2-month**	1.035 (0.899; 1.188)	68.7%	0.97 (0.836; 1.119)	66.1%	1.03 (0.894; 1.19)	65.8%	1.034 (0.893; 1.203)	67.0%				
**4-month**	0.963 (0.824; 1.126)	68.4%	0.994 (0.854; 1.158)	52.9%	1.042 (0.9; 1.202)	71.2%	0.993 (0.855; 1.165)	53.4%				
**C6**												
**2-month**	1.054 (0.917; 1.216)	76.8%	0.989 (0.856; 1.144)	56.1%	1.05 (0.905; 1.218)	73.9%	1.056 (0.911; 1.224)	76.4%	1.057 (0.915; 1.222)	77.3%		
**4-month**	0.961 (0.823; 1.117)	69.3%	0.992 (0.85; 1.161)	54.1%	1.038 (0.892; 1.212)	68.4%	0.994 (0.855; 1.158)	53.2%	1.028 (0.87; 1.204)	62.8%		

Est. – Median of the posterior distribution of incidence rate ratios with 95% compatibility intervals.
Prob. – Proportion of the posterior distribution above or below the null in the direction of the median.

Supplementary Table G2 - Estimates of effects of components, including two-way interactions, on heavy episodic drinking at 2- and 4-month follow-up (imputed data).

	C1		C2		C3		C4		C5		C6	
	**Est.**	**Prob.**	**Est.**	**Prob.**	**Est.**	**Prob.**	**Est.**	**Prob.**	**Est.**	**Prob.**	**Est.**	**Prob.**
**2-month**	0.949 (0.855; 1.057)	82.8%	1.047 (0.94; 1.162)	80.3%	1.069 (0.965; 1.184)	89.8%	0.968 (0.869; 1.079)	72.1%	0.994 (0.897; 1.109)	54.7%	0.976 (0.876; 1.086)	67.2%
**4-month**	0.92 (0.82; 1.031)	92.1%	1.064 (0.951; 1.194)	85.6%	1.043 (0.93; 1.165)	77.0%	1.022 (0.909; 1.139)	64.6%	1.057 (0.944; 1.194)	82.9%	1.072 (0.958; 1.2)	88.3%
**Two-way interactions**												
**C2**												
**2-month**	0.995 (0.85; 1.157)	52.8%										
**4-month**	0.978 (0.833; 1.154)	60.2%										
**C3**												
**2-month**	1.016 (0.877; 1.179)	58.1%	1.12 (0.965; 1.292)	93.2%								
**4-month**	0.959 (0.82; 1.126)	69.6%	1.109 (0.942; 1.307)	89.7%								
**C4**												
**2-month**	0.92 (0.793; 1.069)	86.4%	1.013 (0.874; 1.179)	56.9%	1.034 (0.894; 1.202)	67.3%						
**4-month**	0.939 (0.8; 1.101)	77.5%	1.088 (0.921; 1.28)	83.6%	1.066 (0.901; 1.245)	77.7%						
**C5**												
**2-month**	0.945 (0.811; 1.095)	77.5%	1.041 (0.895; 1.21)	70.5%	1.063 (0.918; 1.227)	79.6%	0.961 (0.827; 1.128)	68.9%				
**4-month**	0.975 (0.826; 1.14)	62.0%	1.126 (0.958; 1.331)	92.4%	1.103 (0.939; 1.293)	88.5%	1.081 (0.919; 1.271)	82.5%				
**C6**												
**2-month**	0.925 (0.794; 1.084)	83.4%	1.023 (0.875; 1.186)	61.5%	1.042 (0.903; 1.207)	71.4%	0.945 (0.812; 1.095)	77.3%	0.97 (0.838; 1.128)	65.3%		
**4-month**	0.983 (0.841; 1.163)	58.1%	1.14 (0.975; 1.338)	95.0%	1.117 (0.954; 1.31)	91.4%	1.094 (0.93; 1.283)	86.4%	1.134 (0.962; 1.342)	92.9%		

Est. – Median of the posterior distribution of incidence rate ratios with 95% compatibility intervals.
Prob. – Proportion of the posterior distribution above or below the null in the direction of the median.

Supplementary Table G3 - Estimates of effects of components, including two-way interactions, on daily portions of fruit and vegetables consumption at 2- and 4-month follow-up (imputed).

	C1		C2		C3		C4		C5		C6	
	**Est.**	**Prob.**	**Est.**	**Prob.**	**Est.**	**Prob.**	**Est.**	**Prob.**	**Est.**	**Prob.**	**Est.**	**Prob.**
**2-month**	0.176 (0.097; 0.246)	>99.9%	0.003 (-0.079; 0.082)	53.2%	0.027 (-0.05; 0.103)	75.4%	0.066 (-0.006; 0.141)	96.3%	-0.011 (-0.092; 0.066)	60.9%	-0.051 (-0.126; 0.025)	90.7%
**4-month**	0.134 (0.059; 0.211)	>99.9%	-0.081 (-0.161; -0.0)	97.5%	0.046 (-0.036; 0.126)	86.6%	-0.064 (-0.145; 0.016)	94.2%	0.031 (-0.051; 0.11)	77.4%	0.025 (-0.06; 0.107)	72.3%
**Two-way interactions**												
**C2**												
**2-month**	0.178 (0.071; 0.282)	>99.9%										
**4-month**	0.054 (-0.062; 0.168)	81.9%										
**C3**												
**2-month**	0.202 (0.095; 0.306)	>99.9%	0.029 (-0.083; 0.145)	69.7%								
**4-month**	0.181 (0.069; 0.288)	99.9%	-0.034 (-0.153; 0.081)	72.1%								
**C4**												
**2-month**	0.241 (0.138; 0.345)	>99.9%	0.069 (-0.04; 0.181)	89.2%	0.092 (-0.01; 0.199)	96.1%						
**4-month**	0.07 (-0.041; 0.185)	88.9%	-0.145 (-0.259; -0.029)	99.3%	-0.018 (-0.128; 0.092)	62.7%						
**C5**												
**2-month**	0.164 (0.054; 0.269)	99.8%	-0.008 (-0.127; 0.107)	55.9%	0.015 (-0.093; 0.122)	61.2%	0.056 (-0.056; 0.161)	84.1%				
**4-month**	0.165 (0.059; 0.271)	99.9%	-0.051 (-0.163; 0.064)	81.1%	0.077 (-0.038; 0.189)	90.6%	-0.034 (-0.142; 0.075)	73.1%				
**C6**												
**2-month**	0.124 (0.016; 0.231)	98.8%	-0.048 (-0.159; 0.059)	80.8%	-0.025 (-0.128; 0.084)	67.9%	0.016 (-0.092; 0.119)	61.3%	-0.062 (-0.174; 0.046)	86.7%		
**4-month**	0.159 (0.043; 0.275)	99.7%	-0.055 (-0.175; 0.055)	83.3%	0.07 (-0.041; 0.183)	89.1%	-0.039 (-0.16; 0.078)	73.8%	0.055 (-0.06; 0.171)	82.0%		

Est. – Median of the posterior distribution of linear effects with 95% compatibility intervals.

Prob. – Proportion of the posterior distribution above or below the null in the direction of the median.

Supplementary Table G4 - Estimates of effects of components, including two-way interactions, on weekly moderate and vigorous physical activity at 2- and 4-month follow-up (imputed).

	C1		C2		C3		C4		C5		C6	
	**Est.**	**Prob.**	**Est.**	**Prob.**	**Est.**	**Prob.**	**Est.**	**Prob.**	**Est.**	**Prob.**	**Est.**	**Prob.**
**2-month**	1.016 (0.921; 1.121)	62.1%	0.952 (0.858; 1.052)	82.8%	1.012 (0.915; 1.121)	59.1%	1.018 (0.916; 1.127)	63.1%	1.018 (0.921; 1.127)	63.3%	1.038 (0.936; 1.151)	75.9%
**4-month**	0.949 (0.853; 1.055)	83.0%	0.979 (0.881; 1.094)	65.4%	1.026 (0.924; 1.139)	68.3%	0.979 (0.883; 1.093)	64.8%	1.015 (0.912; 1.13)	60.4%	1.013 (0.906; 1.13)	59.1%
**Two-way interactions**												
**C2**												
**2-month**	-2.094 (-27.693; 24.495)	56.2%										
**4-month**	2.615 (-27.527; 31.485)	56.7%										
**C3**												
**2-month**	0.725 (-25.715; 28.024)	52.2%	6.166 (-20.825; 33.068)	67.3%								
**4-month**	-4.696 (-34.491; 24.014)	62.3%	6.332 (-23.95; 34.227)	66.4%								
**C4**												
**2-month**	-17.87 (-43.282; 8.777)	90.7%	-12.303 (-38.386; 14.116)	81.9%	-9.775 (-36.686; 18.468)	75.7%						
**4-month**	-2.499 (-30.668; 25.103)	56.8%	8.31 (-19.782; 36.905)	71.9%	0.975 (-28.565; 29.287)	52.7%						
**C5**												
**2-month**	1.09 (-25.969; 27.712)	53.2%	6.789 (-19.705; 34.18)	68.9%	9.417 (-17.344; 35.345)	75.8%	-9.199 (-35.34; 17.242)	75.2%				
**4-month**	0.508 (-28.348; 28.717)	51.4%	11.614 (-16.961; 41.085)	78.3%	4.176 (-24.712; 32.565)	61.2%	6.594 (-23.274; 34.23)	67.5%				
**C6**												
**2-month**	-1.625 (-29.494; 25.641)	54.7%	3.686 (-24.165; 31.035)	60.4%	6.208 (-21.827; 34.443)	67.0%	-12.218 (-39.49; 15.16)	80.8%	6.474 (-19.697; 33.523)	68.5%		
**4-month**	-3.284 (-31.685; 27.8)	58.7%	8.182 (-21.672; 37.202)	70.5%	0.902 (-30.09; 29.524)	52.4%	3.027 (-26.71; 32.038)	58.0%	6.165 (-22.44; 34.359)	66.3%		

Est. – Median of the posterior distribution of linear effects with 95% compatibility intervals.
Prob. – Proportion of the posterior distribution above or below the null in the direction of the median.

Supplementary Table G5 - Estimates of effects of components, including two-way interactions, on smoking cessation at 2- and 4-month follow-up (imputed data).

	C1		C2		C3		C4		C5		C6	
	**Est.**	**Prob.**	**Est.**	**Prob.**	**Est.**	**Prob.**	**Est.**	**Prob.**	**Est.**	**Prob.**	**Est.**	**Prob.**
**2-month**	0.941<br>(0.4; 2.28)	55.5%	1.01<br>(0.433; 2.324)	50.9%	1.426<br>(0.605; 3.313)	79.3%	1.118<br>(0.481; 2.656)	60.3%	0.568<br>(0.243; 1.307)	90.8%	0.769<br>(0.329; 1.773)	73.2%
**4-month**	0.909<br>(0.357; 2.396)	58.0%	1.072<br>(0.429; 2.617)	56.1%	1.706<br>(0.693; 4.284)	87.6%	1.032<br>(0.4; 2.621)	52.6%	0.933<br>(0.357; 2.354)	55.8%	1.17<br>(0.48; 2.978)	63.3%
**Two-way interactions**												
**C2**												
**2-month**	0.953<br>(0.284; 3.197)	53.1%										
**4-month**	0.984<br>(0.276; 3.561)	51.0%										
**C3**												
**2-month**	1.436<br>(0.421; 4.961)	71.9%	1.41<br>(0.404; 4.844)	70.6%								
**4-month**	1.604<br>(0.437; 6.123)	75.9%	1.791<br>(0.481; 6.531)	80.9%								
**C4**												
**2-month**	1.007<br>(0.3; 3.414)	50.4%	1.17<br>(0.348; 3.921)	60.1%	1.712<br>(0.497; 5.922)	80.4%						
**4-month**	0.892<br>(0.242; 3.395)	56.8%	1.121<br>(0.302; 4.14)	56.8%	1.793<br>(0.468; 6.858)	80.3%						
**C5**												
**2-month**	0.576<br>(0.162; 2.057)	80.4%	0.603<br>(0.178; 2.022)	79.5%	0.874<br>(0.262; 2.904)	58.7%	0.581<br>(0.167; 2.003)	80.8%				
**4-month**	0.87<br>(0.228; 3.33)	58.3%	1.06<br>(0.281; 3.89)	53.4%	1.597<br>(0.427; 5.752)	76.0%	0.944<br>(0.239; 3.492)	53.4%				
**C6**												
**2-month**	0.734<br>(0.219; 2.481)	69.4%	0.796<br>(0.239; 2.566)	64.9%	1.138<br>(0.335; 3.782)	58.3%	0.787<br>(0.225; 2.752)	64.7%	0.431<br>(0.124; 1.426)	91.5%		
**4-month**	1.091<br>(0.301; 4.253)	55.2%	1.308<br>(0.378; 4.684)	66.4%	2.049<br>(0.573; 7.406)	86.6%	1.194<br>(0.316; 4.41)	60.6%	1.105<br>(0.3; 4.064)	56.0%		

Est. – Median of the posterior distribution of odds ratios with 95% compatibility intervals.
Prob. – Proportion of the posterior distribution above or below the null in the direction of the median.

Supplementary Table G6 - Estimates of effects of components, including two-way interactions, on number of cigarettes smoked per week at 2- and 4-month follow-up (imputed).

	C1		C2		C3		C4		C5		C6	
	**Est.**	**Prob.**	**Est.**	**Prob.**	**Est.**	**Prob.**	**Est.**	**Prob.**	**Est.**	**Prob.**	**Est.**	**Prob.**
**2-month**	0.978 (0.847; 1.146)	61.0%	1.0 (0.866; 1.155)	50.1%	0.984 (0.848; 1.155)	57.9%	0.99 (0.856; 1.143)	55.1%	0.974 (0.846; 1.133)	64.2%	1.027 (0.879; 1.197)	62.7%
**4-month**	1.018 (0.885; 1.197)	59.7%	1.072 (0.924; 1.262)	81.9%	0.94 (0.771; 1.121)	76.1%	0.98 (0.823; 1.15)	59.8%	1.055 (0.909; 1.233)	75.6%	0.933 (0.779; 1.101)	78.1%
**Two-way interactions**												
**C2**												
**2-month**	0.98 (0.798; 1.213)	57.5%										
**4-month**	1.096 (0.894; 1.369)	80.6%										
**C3**												
**2-month**	0.965 (0.772; 1.204)	62.5%	0.985 (0.794; 1.232)	55.3%								
**4-month**	0.957 (0.753; 1.2)	64.2%	1.01 (0.79; 1.274)	53.3%								
**C4**												
**2-month**	0.97 (0.791; 1.208)	61.1%	0.989 (0.812; 1.216)	54.1%	0.977 (0.787; 1.213)	58.1%						
**4-month**	1.003 (0.801; 1.241)	51.1%	1.048 (0.842; 1.331)	66.2%	0.918 (0.717; 1.172)	75.3%						
**C5**												
**2-month**	0.954 (0.772; 1.196)	66.6%	0.973 (0.801; 1.203)	60.5%	0.96 (0.781; 1.202)	64.5%	0.965 (0.785; 1.186)	63.1%				
**4-month**	1.075 (0.88; 1.354)	75.5%	1.132 (0.908; 1.435)	86.5%	0.992 (0.776; 1.256)	52.8%	1.035 (0.808; 1.301)	61.9%				
**C6**												
**2-month**	1.003 (0.816; 1.26)	51.0%	1.026 (0.831; 1.27)	59.4%	1.012 (0.813; 1.256)	54.1%	1.019 (0.819; 1.257)	56.7%	1.002 (0.813; 1.227)	50.6%		
**4-month**	0.953 (0.754; 1.185)	66.6%	1.001 (0.801; 1.25)	50.4%	0.875 (0.664; 1.124)	84.1%	0.913 (0.711; 1.162)	77.1%	0.986 (0.779; 1.232)	54.7%		

Est. – Median of the posterior distribution of incidence rate ratios with 95% compatibility intervals.

Prob. – Proportion of the posterior distribution above or below the null in the direction of the median.

Supplementary Table G7 - Estimates of effects of components, including two-way interactions, on candy and snacks at 2- and 4-month follow-up (imputed).

	C1		C2		C3		C4		C5		C6	
	**Est.**	**Prob.**	**Est.**	**Prob.**	**Est.**	**Prob.**	**Est.**	**Prob.**	**Est.**	**Prob.**	**Est.**	**Prob.**
**2-month**	0.942 (0.858; 1.038)	88.8%	0.972 (0.882; 1.062)	72.3%	0.99 (0.907; 1.088)	58.4%	1.039 (0.948; 1.14)	79.3%	1.039 (0.953; 1.133)	80.3%	1.008 (0.919; 1.113)	56.4%
**4-month**	0.929 (0.846; 1.017)	94.4%	0.974 (0.88; 1.072)	69.4%	0.867 (0.776; 0.956)	99.8%	0.959 (0.869; 1.063)	79.3%	0.959 (0.869; 1.053)	80.0%	1.058 (0.962; 1.163)	87.6%
**Two-way interactions**												
**C2**												
**2-month**	0.917 (0.794; 1.035)	90.9%										
**4-month**	0.905 (0.785; 1.034)	92.7%										
**C3**												
**2-month**	0.934 (0.818; 1.068)	83.4%	0.963 (0.841; 1.096)	70.1%	1.029 (0.896; 1.18)	67.5%						
**4-month**	0.802 (0.696; 0.922)	99.9%	0.843 (0.728; 0.971)	99.1%	0.831 (0.72; 0.959)	99.4%						
**C4**												
**2-month**	0.979 (0.863; 1.109)	62.0%	1.009 (0.876; 1.156)	55.1%	0.999 (0.883; 1.131)	50.6%						
**4-month**	0.889 (0.779; 1.021)	95.0%	0.938 (0.8; 1.065)	81.8%	0.826 (0.722; 0.949)	99.7%						
**C5**												
**2-month**	0.977 (0.869; 1.117)	65.1%	1.007 (0.889; 1.143)	54.4%	1.028 (0.909; 1.171)	66.9%	1.08 (0.95; 1.216)	88.5%				
**4-month**	0.888 (0.781; 1.022)	94.9%	0.935 (0.807; 1.07)	83.9%	0.832 (0.719; 0.943)	99.9%	0.92 (0.804; 1.059)	88.6%				
**C6**												
**2-month**	0.95 (0.834; 1.095)	77.6%	0.981 (0.858; 1.106)	60.9%	0.998 (0.888; 1.136)	51.0%	1.046 (0.924; 1.195)	75.1%	1.046 (0.922; 1.194)	76.4%		
**4-month**	0.984 (0.857; 1.121)	59.8%	1.03 (0.902; 1.178)	66.1%	0.916 (0.791; 1.058)	89.4%	1.013 (0.88; 1.184)	57.0%	1.015 (0.893; 1.155)	59.0%		

Est. – Median of the posterior distribution of incidence rate ratios with 95% compatibility intervals.
Prob. – Proportion of the posterior distribution above or below the null in the direction of the median.

Supplementary Table G8 - Estimates of effects of components, including two-way interactions, on weekly sugary drinks consumption at 2- and 4-month follow-up (imputed).

	C1		C2		C3		C4		C5		C6	
	**Est.**	**Prob.**	**Est.**	**Prob.**	**Est.**	**Prob.**	**Est.**	**Prob.**	**Est.**	**Prob.**	**Est.**	**Prob.**
**2-month**	0.911 (0.758; 1.092)	83.7%	0.95 (0.794; 1.136)	70.8%	1.03 (0.851; 1.24)	61.8%	0.943 (0.782; 1.133)	73.2%	0.952 (0.786; 1.138)	69.8%	1.172 (0.959; 1.419)	93.9%
**4-month**	1.017 (0.811; 1.258)	56.3%	0.948 (0.79; 1.167)	69.4%	0.935 (0.756; 1.145)	74.5%	0.865 (0.704; 1.04)	93.9%	1.053 (0.849; 1.313)	67.8%	1.065 (0.865; 1.351)	72.2%
**Two-way interactions**												
**C2**												
**2-month**	0.864 (0.663; 1.121)	86.1%										
**4-month**	0.969 (0.712; 1.279)	58.3%										
**C3**												
**2-month**	0.94 (0.726; 1.212)	68.5%	0.977 (0.757; 1.274)	56.5%								
**4-month**	0.95 (0.702; 1.276)	63.3%	0.884 (0.669; 1.194)	79.0%								
**C4**												
**2-month**	0.859 (0.661; 1.115)	87.3%	0.894 (0.686; 1.174)	79.4%	0.974 (0.732; 1.277)	57.3%						
**4-month**	0.882 (0.652; 1.147)	81.4%	0.82 (0.625; 1.081)	92.0%	0.802 (0.609; 1.092)	92.1%						
**C5**												
**2-month**	0.868 (0.652; 1.13)	84.5%	0.899 (0.691; 1.191)	77.4%	0.976 (0.754; 1.28)	57.1%	0.896 (0.681; 1.177)	78.7%				
**4-month**	1.071 (0.777; 1.468)	66.2%	0.998 (0.748; 1.371)	50.4%	0.979 (0.736; 1.336)	55.6%	0.912 (0.672; 1.208)	73.2%				
**C6**												
**2-month**	1.064 (0.823; 1.4)	67.9%	1.114 (0.843; 1.449)	77.5%	1.204 (0.916; 1.588)	90.3%	1.11 (0.835; 1.429)	77.1%	1.114 (0.84; 1.448)	77.2%		
**4-month**	1.089 (0.796; 1.456)	70.5%	1.01 (0.768; 1.373)	52.6%	1.0 (0.737; 1.33)	50.1%	0.925 (0.684; 1.212)	71.0%	1.123 (0.838; 1.515)	80.0%		

Est. – Median of the posterior distribution of incidence rate ratios with 95% compatibility intervals.
Prob. – Proportion of the posterior distribution above or below the null in the direction of the median.

Supplementary Table G9 - Estimates of effects of components, including two-way interactions, on body mass index at 2- and 4-month follow-up (imputed).

	C1		C2		C3		C4		C5		C6	
	**Est.**	**Prob.**	**Est.**	**Prob.**	**Est.**	**Prob.**	**Est.**	**Prob.**	**Est.**	**Prob.**	**Est.**	**Prob.**
**2-month**	-0.001 (-0.42; 0.404)	50.3%	0.141 (-0.293; 0.57)	74.2%	0.059 (-0.348; 0.474)	61.2%	-0.162 (-0.597; 0.274)	77.0%	-0.238 (-0.695; 0.176)	86.4%	-0.113 (-0.537; 0.317)	69.4%
**4-month**	-0.071 (-0.502; 0.35)	62.5%	0.203 (-0.243; 0.646)	81.6%	0.065 (-0.355; 0.498)	61.7%	-0.228 (-0.673; 0.221)	84.3%	-0.234 (-0.693; 0.197)	85.0%	-0.148 (-0.586; 0.295)	73.9%
**Two-way interactions**												
**C2**												
**2-month**	0.135 (-0.474; 0.771)	66.4%										
**4-month**	0.128 (-0.495; 0.776)	65.3%										
**C3**												
**2-month**	0.052 (-0.564; 0.66)	56.2%	0.2 (-0.378; 0.789)	75.2%								
**4-month**	-0.015 (-0.647; 0.617)	51.7%	0.267 (-0.33; 0.876)	81.1%								
**C4**												
**2-month**	-0.185 (-0.783; 0.417)	72.1%	-0.037 (-0.628; 0.586)	54.9%	-0.113 (-0.714; 0.472)	64.3%						
**4-month**	-0.322 (-0.937; 0.299)	83.8%	-0.036 (-0.648; 0.597)	54.6%	-0.173 (-0.794; 0.433)	70.9%						
**C5**												
**2-month**	-0.232 (-0.851; 0.372)	77.4%	-0.07 (-0.674; 0.526)	59.3%	-0.194 (-0.812; 0.407)	73.1%	-0.404 (-0.999; 0.194)	90.7%				
**4-month**	-0.296 (-0.939; 0.324)	82.4%	-0.003 (-0.622; 0.617)	50.3%	-0.184 (-0.834; 0.446)	71.7%	-0.465 (-1.071; 0.144)	92.9%				
**C6**												
**2-month**	-0.108 (-0.703; 0.48)	63.6%	0.034 (-0.558; 0.644)	54.5%	-0.044 (-0.655; 0.541)	55.5%	-0.252 (-0.863; 0.359)	78.9%	-0.333 (-0.922; 0.252)	86.7%		
**4-month**	-0.209 (-0.829; 0.406)	74.6%	0.067 (-0.548; 0.692)	58.5%	-0.073 (-0.705; 0.532)	59.2%	-0.355 (-0.984; 0.274)	86.4%	-0.357 (-0.968; 0.248)	87.7%		

Est. – Median of the posterior distribution of linear effects with 95% compatibility intervals.

Prob. – Proportion of the posterior distribution above or below the null in the direction of the median.

Supplementary Table G10- Estimates of effects of components, including two-way interactions, on perceived stress at 2- and 4-month follow-up (imputed).

	C1		C2		C3		C4		C5		C6	
	**Est.**	**Prob.**	**Est.**	**Prob.**	**Est.**	**Prob.**	**Est.**	**Prob.**	**Est.**	**Prob.**	**Est.**	**Prob.**
**2-month**	0.046 (-0.135; 0.248)	68.6%	-0.002 (-0.196; 0.197)	50.9%	-0.059 (-0.265; 0.142)	71.9%	-0.253 (-0.444; -0.061)	99.6%	-0.152 (-0.348; 0.059)	92.2%	-0.12 (-0.299; 0.078)	88.5%
**4-month**	-0.038 (-0.27; 0.192)	63.0%	0.088 (-0.136; 0.305)	77.3%	-0.103 (-0.313; 0.111)	81.9%	0.029 (-0.2; 0.243)	60.3%	-0.197 (-0.399; 0.018)	96.4%	0.018 (-0.182; 0.249)	56.7%
**Two-way interactions**												
**C2**												
**2-month**	0.047 (-0.219; 0.322)	63.1%										
**4-month**	0.053 (-0.289; 0.357)	62.8%										
**C3**												
**2-month**	-0.014 (-0.278; 0.283)	53.9%	-0.062 (-0.352; 0.24)	65.4%								
**4-month**	-0.146 (-0.451; 0.183)	79.4%	-0.015 (-0.315; 0.274)	53.9%								
**C4**												
**2-month**	-0.204 (-0.473; 0.074)	92.9%	-0.258 (-0.529; 0.038)	95.4%	-0.317 (-0.592; -0.008)	97.8%						
**4-month**	-0.011 (-0.319; 0.294)	52.8%	0.116 (-0.204; 0.422)	75.3%	-0.07 (-0.393; 0.218)	66.7%						
**C5**												
**2-month**	-0.106 (-0.371; 0.201)	76.3%	-0.156 (-0.426; 0.143)	84.6%	-0.209 (-0.492; 0.074)	92.6%	-0.401 (-0.683; -0.134)	99.9%				
**4-month**	-0.23 (-0.55; 0.059)	93.9%	-0.113 (-0.405; 0.217)	76.9%	-0.296 (-0.605; -0.003)	97.6%	-0.166 (-0.472; 0.118)	87.0%				
**C6**												
**2-month**	-0.074 (-0.339; 0.233)	69.0%	-0.119 (-0.393; 0.156)	80.0%	-0.177 (-0.443; 0.094)	89.5%	-0.369 (-0.634; -0.113)	99.8%	-0.271 (-0.537; 0.017)	96.7%		
**4-month**	-0.021 (-0.31; 0.302)	55.2%	0.113 (-0.215; 0.407)	76.0%	-0.084 (-0.376; 0.24)	70.8%	0.045 (-0.262; 0.372)	60.9%	-0.174 (-0.452; 0.112)	88.1%		

Est. – Median of the posterior distribution of linear effects with 95% compatibility intervals.
Prob. – Proportion of the posterior distribution above or below the null in the direction of the median.

Supplementary Table G11 - Estimates of effects of components, including two-way interactions, on PROMIS at 4-month follow-up (imputed).

	C1		C2		C3		C4		C5		C6	
	**Est.**	**Prob.**	**Est.**	**Prob.**	**Est.**	**Prob.**	**Est.**	**Prob.**	**Est.**	**Prob.**	**Est.**	**Prob.**
**4-month**	-0.154<br>(-0.682; 0.349)	71.8%	-0.16<br>(-0.734; 0.383)	71.7%	0.235<br>(-0.301; 0.796)	80.1%	0.165<br>(-0.384; 0.778)	71.6%	0.016<br>(-0.527; 0.576)	52.3%	-0.093<br>(-0.599; 0.401)	64.3%
**Two-way interactions**												
**C2**												
**4-month**	-0.322<br>(-1.078; 0.444)	79.6%										
**C3**												
**4-month**	0.071<br>(-0.67; 0.865)	57.6%	0.079<br>(-0.718; 0.802)	58.6%								
**C4**												
**4-month**	-0.001<br>(-0.74; 0.902)	50.1%	0.006<br>(-0.748; 0.804)	50.6%	0.393<br>(-0.332; 1.243)	84.1%						
**C5**												
**4-month**	-0.132<br>(-0.879; 0.555)	64.2%	-0.141<br>(-0.913; 0.609)	64.2%	0.244<br>(-0.492; 1.109)	74.0%	0.193<br>(-0.611; 0.977)	67.6%				
**C6**												
**4-month**	-0.248<br>(-1.013; 0.485)	74.0%	-0.239<br>(-1.083; 0.453)	74.4%	0.137<br>(-0.588; 0.907)	63.7%	0.074<br>(-0.665; 0.864)	57.6%	-0.064<br>(-0.738; 0.72)	57.5%		

Est. – Median of the posterior distribution of linear effects with 95% compatibility intervals.

Prob. – Proportion of the posterior distribution above or below the null in the direction of the median.
