# Supplementary material for: Effectiveness of the Components of a Digital Multiple Health Behavior Change Intervention Among Individuals Seeking Help Online (Coach): Factorial Randomized Trial
Source: J Med Internet Res. 2026 Apr 2;28:e88881. doi: 10.2196/88881 (PMC13087559; doi:10.2196/88881)
Supplement: Multimedia Appendix 9 [file jmir_v28i1e88881_app9.pdf]

MULTIMEDIA APPENDIX 9 – ESTIMATES OF EFFECTS OF NUMBER OF COMPONENTS

In this appendix, post-hoc exploratory analyses are presented which estimate the effects of number of components participants had access to on primary and secondary outcomes. Analyses with complete case and imputed data are presented in Supplementary Tables H1 through H11.

Statistical analysis:
We modlled outcomes conditional on number of components using multilevel regression models with covariates for time-by-number of componet interactions and participant level adaptative intercepts. All models were adjusted for presence/absence of all components, as well as baseline measures of age, sex, importance, confidence, and know-how. Primary outcomes, stress, and weekly number of cigarettes smoked were additionally adjusted for their respective measures at baseline. BMI, sugary drinks, and sweets and snacks were adjusted for baseline MVPA minutes per week and average intake of fruit and vegetables per day. QoL was adjusted for perceived stress at baseline.

SUMMARY

In summary, the relatively strongest evidence for effects was found for increasing the module count with regards to fruit and vegetables and stress at the 2-month follow-up. There was also evidence for reduced candy and snacks consumption with increased module count at the 4-month follow-up interval.

TABLES

TOTAL WEEKLY ALCOHOL CONSUMPTION

Supplementary Table H1 - Estimates of effects of number of components on total weekly alcohol consumption.

	Est.	Prob.
**Complete-case**		
2-month	1.01 (0.97; 1.06)	68.9%
4-month	1.01 (0.95; 1.06)	58.1%
**Imputed data**		
2-month	1.01 (0.97; 1.05)	65.4%
4-month	0.99 (0.95; 1.04)	61.1%
**Est.** – Median of the posterior distribution of incidence rate ratios with 95% compatibility intervals. **Prob.** – Proportion of the posterior distribution above or below the null in the direction of the median.		

FREQUENCY OF HEAVY EPISODIC DRINKING

Supplementary Table H2 - Estimates of effects of number of components on frequency of heavy episodic drinking.

	**Est.**	**Prob.**
**Complete-case**		
2-month	1.00 0.96; 1.04)	52.0%
4-month	1.04 (0.99; 1.09)	93.2%
**Imputed data**		
2-month	1.00 (0.96; 1.04)	50.5%
4-month	1.03 (0.98; 1.08)	87.6%

Est. – Median of the posterior distribution of incidence rate ratios with 95% compatibility intervals.
Prob. – Proportion of the posterior distribution above or below the null in the direction of the median.

AVERAGE DAILY CONSUMPTION OF FRUIT AND VEGETABLES

Supplementary Table H3 - Estimates of effects of number of components on fruit and vegetables consumption.

	**Est.**	**Prob.**
**Complete-case**		
2-month	0.032 (0.001; 0.063)	97.7%
4-month	0.016 (-0.018; 0.051)	81.8%
**Imputed data**		
2-month	0.035 (0.004; 0.066)	98.6%
4-month	0.015 (-0.018; 0.048)	81.4%

Est. – Median of the posterior distribution of linear effects with 95% compatibility intervals.
Prob. – Proportion of the posterior distribution above or below the null in the direction of the median.

WEEKLY SUGARY DRINKS CONSUMPTION

Supplementary Table H4 - Estimates of effects of number of components on weekly sugary drinks consumption.

	**Est.**	**Prob.**
**Complete-case**		
2-month	0.98 (0.91; 1.06)	67.5%
4-month	0.99 (0.90; 1.08)	63.3%
**Imputed data**		
2-month	0.99 (0.91; 1.08)	60.0%
4-month	0.98 (0.90; 1.06)	69.5%

**Est.** – Median of the posterior distribution of incidence rate ratios with 95% compatibility intervals.
**Prob.** – Proportion of the posterior distribution above or below the null in the direction of the median.

WEEKLY MODERATE TO VIGOROUS PHYSICAL ACTIVITY

Supplementary Table H5 - Estimates of effects of number of components on weekly moderate to vigorous physical activity.

	Est.	Prob.
**Complete-case**		
2-month	0.79 (-7.41; 8.94)	57.2%
4-month	3.40 (-5.6; 12.6)	76.9%
**Imputed data**		
2-month	-0.83 (-8.61; 6.99)	58.2%
4-month	1.60 (-7.29; 9.92)	63.9%
**Est.** – Median of the posterior distribution of linear effects with 95% compatibility intervals. **Prob.** – Proportion of the posterior distribution above or below the null in the direction of the median.		

SMOKING CESSATION

Supplementary Table H6 - Estimates of effects of number of components on smoking cessation.

	Est.	Prob.
**Complete-case**		
2-month	0.94 (0.67; 1.31)	63.6%
4-month	1.23 (0.86; 1.78)	87.0%
**Imputed data**		
2-month	0.92 (0.64; 1.31)	68.5%
4-month	1.11 (0.77; 1.60)	70.7%
**Est.** – Median of the posterior distribution of odds ratios with 95% compatibility intervals. **Prob.** – Proportion of the posterior distribution above or below the null in the direction of the median.		

NUMBER OF CIGARETTES SMOKED PER WEEK

Supplementary Table H7 - Estimates of effects of number of components on number of cigarettes smoked per week.

	Est.	Prob.
**Complete-case**		
2-month	1.01 (0.94; 1.09)	63.5%
4-month	0.99 (0.91; 1.08)	56.9%
**Imputed data**		
2-month	0.99 (0.93; 1.06)	59.8%

4-month	1.00 (0.93; 1.07)	52.1%
**Est.** – Median of the posterior distribution of incidence rate ratios with 95% compatibility intervals.<br>**Prob.** – Proportion of the posterior distribution above or below the null in the direction of the median.		

WEEKLY PORTIONS OF CANDY AND SNACKS

Supplementary Table H8 - Estimates of effects of number of components on weekly portions of candy and snacks.

	Est.	Prob.
**Complete-case**		
2-month	0.99 (0.95; 1.02)	80.0%
4-month	0.95 (0.91; 0.99)	99.6%
**Imputed data**		
2-month	1.00 (0.96; 1.03)	54.6%
4-month	0.96 (0.92; 1.00)	98.7%
**Est.** – Median of the posterior distribution of incidence rate ratios with 95% compatibility intervals.<br>**Prob.** – Proportion of the posterior distribution above or below the null in the direction of the median.		

BODY MASS INDEX

Supplementary Table H9 - Estimates of effects of number of components on body mass index.

	Est.	Prob.
**Complete-case**		
2-month	-0.053 (-0.224; 0.113)	72.1%
4-month	-0.067 (-0.243; 0.103)	77.0%
**Imputed data**		
2-month	-0.053 (-0.22; 0.135)	73.1%
4-month	-0.073 (-0.25; 0.115)	79.9%
**Est.** – Median of the posterior distribution of linear effects with 95% compatibility intervals.<br>**Prob.** – Proportion of the posterior distribution above or below the null in the direction of the median.		

PERCEIVED STRESS

Supplementary Table H10 - Estimates of effects of number of components on perceived stress.

	Est.	Prob.
**Complete-case**		
2-month	-0.10 (-0.18; -0.02)	99.1%
4-month	-0.04 (-0.13; 0.05)	80.8%
**Imputed data**		

2-month	-0.09 (-0.17; -0.003)	97.9%
4-month	-0.03 (-0.12; 0.06)	76.3%
**Est.** – Median of the posterior distribution of linear effects with 95% compatibility intervals.<br>**Prob.** – Proportion of the posterior distribution above or below the null in the direction of the median.		

QUALITY OF LIFE

Supplementary Table H11 - Estimates of effects of number of components on quality of life.

	Est.	Prob.
**Complete-case**		
4-month	0.07 (-0.17; 0.30)	72.2%
**Imputed data**		
4-month	-0.001 (-0.22; 0.24)	50.4%
**Est.** – Median of the posterior distribution of linear effects with 95% compatibility intervals.<br>**Prob.** – Proportion of the posterior distribution above or below the null in the direction of the median.		
